# Performance of mechanically sheared DNA in multiplexed Oxford Nanopore sequencing for Salmonella Typhi genomic surveillance

**DOI:** 10.1099/mgen.0.001805

**Published:** 2026-07-27

**Authors:** Hsu Thinzar Maung, Masamoto Morita, Su Myat Han, Hiroyuki Mishima, Chisa Koga, Jan Wendzl C. Evangelista, Mark John Girasol, Shuichi Suzuki, Koh-ichiro Yoshiura, Toshio Kodama, Chris Smith, Hirotaka Hiyoshi

**Affiliations:** 1 School of Tropical Medicine and Global Health (TMGH), Nagasaki University, Nagasaki, Japan; 2 Department of Bacteriology, Institute of Tropical Medicine (NEKKEN), Nagasaki University, Nagasaki, Japan; 3 Department of Bacteriology I, National Institute of Infectious Diseases (NIID), Japan Institute for Health Security, Tokyo, Japan; 4 Department of Human Genetics, Atomic Bomb Disease Institute (GENKEN), Nagasaki University, Nagasaki, Japan; 5 San Lazaro Hospital-Nagasaki University Research and Training Collaborating Center (SLH-NU), Manila, Philippines; 6 Department of Molecular Bacteriology, Research Institute for Microbial Diseases (RIMD), the University of Osaka, Osaka, Japan; 7 Department of Clinical Research, Faculty of Infectious and Tropical Diseases, London School of Hygiene and Tropical Medicine (LSHTM), London, UK

**Keywords:** genomic surveillance, mechanical DNA shearing, multiplex sequencing, Oxford Nanopore Technologies, *Salmonella *Typhi, typhoid

## Abstract

Pathogen genomic surveillance is essential for monitoring high-risk lineages and antimicrobial resistance in *Salmonella enterica* serovar Typhi (*S*. Typhi), yet implementing whole-genome sequencing in sentinel hospital laboratories remains challenging. We evaluated whether mechanically sheared DNA, paired with high accuracy or super accuracy (SUP) basecalling, enables reliable Oxford Nanopore Technologies (ONT)-only sequencing for multiplexed *S*. Typhi genomic surveillance. Genomic DNA from three benchmark strains (Ty2, Ty42 and Ty43) was either mechanically sheared to ~15 kb or left unsheared and sequenced in a controlled 6-plex run. The optimized workflow was subsequently evaluated in a 24-plex field run that included 3 benchmark strains and 21 clinical isolates from a sentinel hospital in Manila, Philippines. Both runs were performed using the ONT Ligation Sequencing Kit with Native Barcoding 24 V14 (SQK-NBD114.24) and R10.4.1 flow cells (FLO-MIN114). Assembly metrics, single-nucleotide variation (SNV) concordance, core-genome MLST and downstream functional outputs were compared across DNA preparations and basecalling modes. In the 6-plex run, sheared libraries consistently achieved >1,000× pre-filtered coverage across all strains, whereas unsheared libraries showed highly variable depths and reduced stability after downsampling. When paired with SUP basecalling, sheared datasets produced assemblies comparable to Illumina and hybrid references across both structural accuracy and functional outputs. In the 24-plex run, all samples achieved >100× coverage, and 18 of the 22 retained *S*. Typhi genomes (81.8%) yielded fully circularized assemblies after exclusion of 2 non-*S*. Typhi isolates. Serotype prediction, genotyping and *in silico* antimicrobial resistance predictions remained concordant with the corresponding 6-plex benchmark results. These findings demonstrate that mechanical DNA shearing improves coverage uniformity under multiplexed conditions and, together with SUP basecalling, supports ONT-only *S*. Typhi genome reconstruction for routine genomic surveillance and broad phylogenetic contextualization. However, for fine-scale transmission analysis and high-resolution SNV interpretation, short-read or hybrid sequencing remains important. Overall, this approach provides a scalable framework for high-multiplex *S*. Typhi genomic surveillance in sentinel laboratory settings.

**Impact Statement**

Genomic surveillance for typhoid remains largely centralized in reference laboratories, although sentinel hospitals are often where cases are first detected, and timely information could most directly inform public health response. Oxford Nanopore Technologies (ONT) offers a potential route towards more decentralized sequencing, but implementation requires workflows that remain reliable when multiple isolates are multiplexed on one flow cell. This study provides pilot evidence that a simple workflow modification, mechanical DNA shearing to ~15 kb combined with super accuracy basecalling, can improve coverage consistency in high-multiplex ONT sequencing while preserving surveillance-relevant outputs for *Salmonella enterica* serovar Typhi. The public health importance of this work lies in demonstrating an implementation-oriented ONT-only workflow for resource-constrained laboratories entering genomic surveillance. Rather than relying on high-throughput GridION or PromethION platforms or high-performance computing environments, this study used a portable MinION Mk1B, standard consumer hardware and web-based platforms for downstream analysis. These findings support a linked surveillance model in which sentinel hospitals generate high-quality sequence data locally, while reference laboratories provide analytical support, validation, quality assurance and integration into national surveillance systems. Such an approach could shift sentinel hospitals from passive sample collection sites to active genomic surveillance partners, while maintaining the interpretive support needed for public health action.

## Data Summary

1. Raw sequencing data for all isolates have been deposited in the DDBJ Sequence Read Archive under BioProject accession PRJDB40029. Individual DRA run accession numbers (DRR911410–DRR911446) are provided in Table S1, available in the online Supplementary Material.
2. Annotated assemblies generated in this study have been deposited in DDBJ/EMBL/GenBank under accession numbers (BAAJZI010000001–BAAKAV010000001), with complete accession numbers available in Table S1.
3. Supporting analysis files used for data analysis and figure generation are available at Zenodo (https://doi.org/10.5281/zenodo.20746114).

The authors confirm that all supporting data, code and protocols have been provided within the article or through supplementary data files.

## Introduction

Typhoid fever, caused by *Salmonella enterica* serovar Typhi (*S*. Typhi), remains a major public health threat in many low- and middle-income countries (LMICs) [1, 2]. Despite global progress in vaccination and sanitation, endemic transmission persists in South and Southeast Asia, sub-Saharan Africa and parts of Oceania [1, 2]. Increasing rates of multidrug-resistant and extensively drug-resistant *S*. Typhi have made timely detection and surveillance an urgent public health priority [3, 4].

Genomic surveillance has become central to detecting outbreaks, tracking antimicrobial resistance and identifying the emergence and spread of high-risk lineages [3–5]. Yet many countries, especially LMICs, remain heavily dependent on centralized sequencing facilities that require long-distance sample transport, specialized personnel, cold-chain stability and reliable computational infrastructure [6–8]. These constraints delay data generation and often limit the integration of genomic results into real-time clinical care or public health action. The COVID-19 pandemic underscored the vulnerability of this centralized model, when global disruptions in capacity further widened the gap between countries with operational genomic surveillance and those without [6–10]. Decentralized sequencing, at sentinel hospitals, offers a practical alternative [6, 8]. Generating genomic data closer to the point of care can accelerate lineage tracking, detect shifts in circulating genotypes and support antimicrobial resistance (AMR)-informed clinical management. Nevertheless, implementation remains challenging. Laboratory equipment and reagent costs remain high, and many frontline laboratories lack personnel with experience in bioinformatics workflows [6, 8, 11, 12].

Portable long-read platforms such as Oxford Nanopore Technologies (ONT) offer advantages for decentralized pathogen sequencing, including real-time analysis and lower operational requirements than conventional short-read platforms [13–17]. Long reads can also improve structural resolution by spanning repetitive regions and supporting reconstruction of complete bacterial replicons, including chromosomes and plasmids that may carry AMR determinants [13, 16]. Recent chemistry advances (e.g. R10.4.1 flow cells and Q20+ kits), along with improved basecalling models, have enabled more complete whole genome assemblies and substantially narrowed the accuracy gap with short-read sequencing [13, 14, 16]. Despite these advances, challenges remain in high-multiplex surveillance applications.

The first challenge is coverage uniformity. Increasing the number of barcoded samples per flow cell can lead to unequal read distribution, substantial depth variation and reduced reliability for downstream analyses if some samples receive insufficient coverage [18, 19]. High-molecular-weight DNA preparations with heterogeneous fragment sizes may further exacerbate this imbalance due to variable loading efficiency and stochastic fragmentation dynamics during sequencing [19]. The minimum coverage requirement also varies depending on the intended genomic output. Previous studies have shown that accurate serotype prediction can be achieved at ~30× to 50× coverage, while AMR or virulence gene detection may also perform well at lower coverage when appropriate tools and thresholds are used [17, 20, 21]. Other ONT-only bacterial assembly studies have reported high-quality genome reconstruction at ~75× coverage under optimized conditions [22]. However, assembly comparison, single-nucleotide variation (SNV) concordance, cgMLST profiling and phylogenetic contextualization benefit from more conservative and consistent coverage. For high-quality bacterial genome reconstruction, ~100× ONT coverage has been recommended as a minimum depth [13]; therefore, this study used 100× as a conservative standardized benchmarking target rather than a universal surveillance requirement. DNA shearing has been proposed as a strategy to normalize fragment length distributions, improve pore loading consistency and enhance coverage uniformity [23, 24]; however, its value in multiplexed whole-genome sequencing workflows for bacterial surveillance has not been systematically evaluated.

The second challenge is per-base accuracy and its interaction with basecalling mode. Although super accuracy (SUP) basecalling offers higher per-base accuracy than high accuracy (HAC), HAC remains attractive for its lower computational demands and faster turnaround time [13, 25–27]. Importantly, recent reports have shown that these residual errors are not uniform but are heavily strain-dependent. Lineage-specific DNA methylation motifs and restriction-modification systems can systematically confound basecalling models, causing localized consensus distortions that vary between individual isolates even within the same serovar [28]. These issues are particularly relevant for *S*. Typhi, a highly clonal pathogen where small differences in SNV or cgMLST profiles may influence lineage interpretation. However, the performance of these different basecalling modes when using sheared DNA, under multiplexed, real-world surveillance conditions, remains unclear.

In this study, we evaluated an ONT-based workflow designed for decentralized *S*. Typhi genomic surveillance suitable for laboratories operating under resource constraints. We benchmarked mechanically sheared DNA and unsheared DNA preparations across three *S*. Typhi strains using HAC and SUP basecalling on R10.4.1 flow cells with the ONT Ligation Native Barcoding Kit, assessing read quality, assembly accuracy, SNV concordance, cgMLST profiles, genotyping, serotype prediction and AMR prediction using hybrid (Illumina+ONT) assemblies as ground truth. To assess real-world applicability, we applied the optimized sheared DNA workflow in a 24-plex run that included the 3 benchmark strains and 21 clinical isolates from a sentinel hospital in Manila, Philippines.

## Methods

### Bacterial isolates

ONT sequencing performance was evaluated using three *S*. Typhi benchmark strains obtained from the National Institute of Infectious Diseases (NIID), Tokyo, Japan: Ty2 (a reference strain), Ty42 and Ty43 (clinical isolates).

In addition, 21 clinical isolates initially identified by MALDI-TOF as *S*. Typhi from San Lazaro Hospital (SLH), Manila, were included for field validation. These isolates were originally collected as part of a Community-Acquired Bacteremia surveillance study at SLH, the national infectious disease referral hospital and a key sentinel site for infectious disease surveillance in the Philippines [29]. All isolates were archived at −80 °C, and no previous genomic sequencing had been conducted for these isolates.

Species identities were reconfirmed before sequencing using the *S. enterica* PCR assay described by Hirose *et al*. [30]. Of the 21 SLH clinical isolates, 19 were confirmed as *S*. Typhi. Two isolates were identified as non-Typhi isolates by both PCR and *in silico* analysis and were excluded from downstream analyses (Table S4). Full isolate metadata are provided in Table S1.

### Study design

The study comprised two sequencing runs:

1. **Run 1 (6-plex):** Controlled benchmarking comparing sheared and unsheared DNA across HAC and SUP basecalling modes.
2. **Run 2 (24-plex):** Field-level evaluation of the optimized workflow using sheared DNA exclusively.

**Fig. 1.**
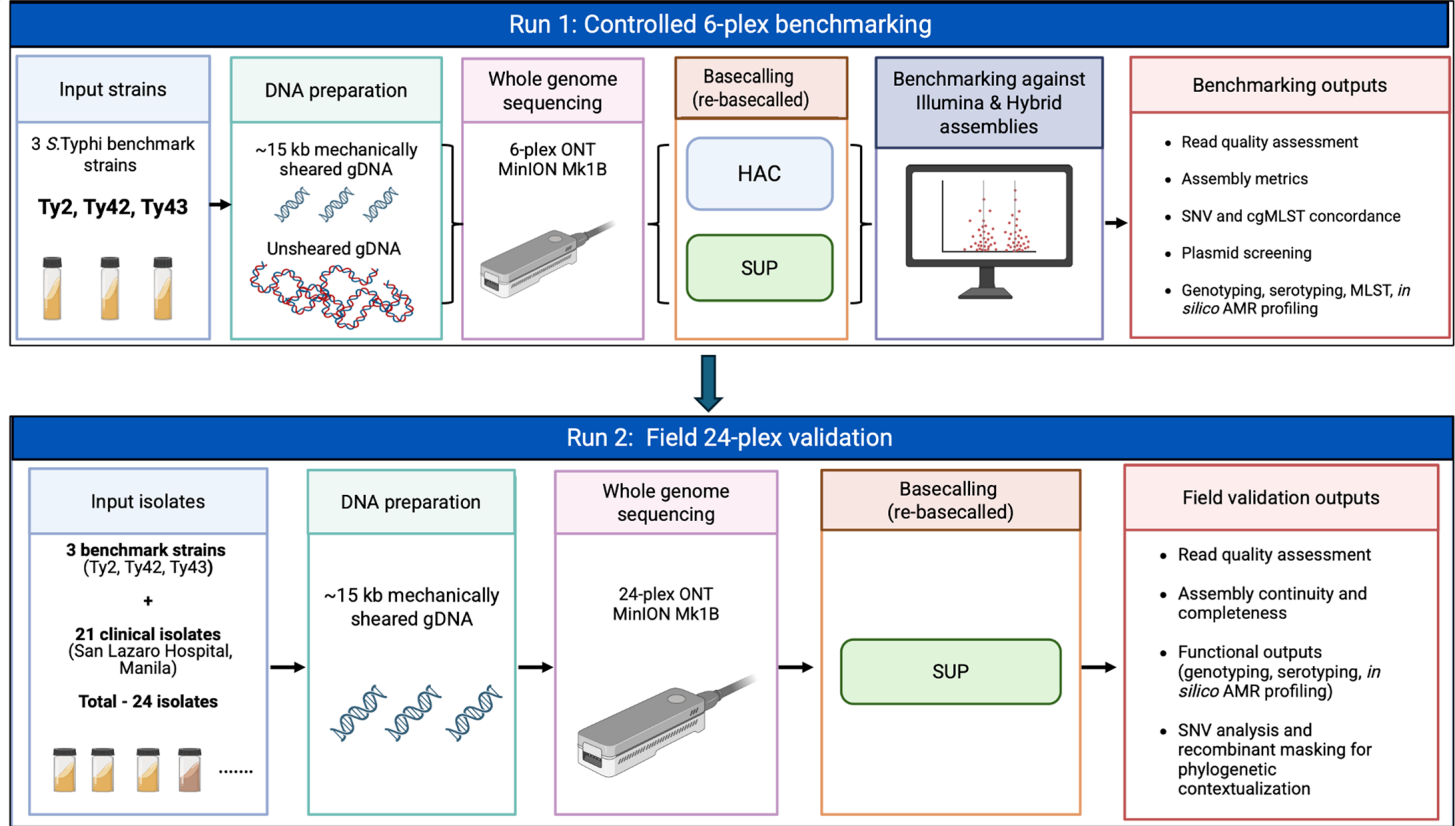
Overview of the two-stage ONT sequencing workflow used for controlled benchmarking and field validation. Run 1 compared mechanically sheared genomic DNA (gDNA; ~15 kb) and unsheared high-molecular-weight gDNA from three *S*. Typhi benchmark strains (Ty2, Ty42 and Ty43) in a controlled 6-plex ONT sequencing run. Libraries were prepared using the ONT Ligation Sequencing Kit with Native Barcoding 24 V14 (SQK-NBD114.24) and sequenced on an R10.4.1 MinION flow cell. Data were rebasecalled using HAC and SUP models and evaluated against Illumina and hybrid assemblies. Run 2 was a 24-plex field validation run containing the 3 benchmark strains and 21 clinical isolates from SLH, Manila, using mechanically sheared gDNA and SUP basecalling. HAC, high accuracy basecalling; SUP, super accuracy basecalling; ONT, Oxford Nanopore Technologies; gDNA, genomic DNA; SNV, single-nucleotide variant; cgMLST, core-genome MLST; AMR, antimicrobial resistance.

An overview of the study design is shown in Fig. 1.

### DNA extraction and the shearing process

All isolates were cultured overnight in 20 ml Luria–Bertani (LB) broth at 37 °C with aeration [31]. Genomic DNA (gDNA) from *S*. Typhi was extracted using the Qiagen gDNA Buffer Kit with Genomic-tip 500 G^−1^, following the manufacturer’s instructions. DNA quantity was measured using Qubit^™^ fluorometric assays (Thermo Fisher Scientific), and DNA purity was assessed by a NanoDrop spectrophotometer (Thermo Fisher Scientific).

For benchmarking, each extracted gDNA was divided into two preparations:

- unsheared high-molecular-weight gDNA and
- mechanically sheared gDNA generated using Covaris g-TUBEs^™^ (Covaris)

DNA integrity and fragment size distributions were then assessed using the Agilent TapeStation gDNA ScreenTape assay, with DNA Integrity Number (DIN) values generated by the TapeStation software. High-molecular-weight gDNA (DIN 8.0–9.8) was diluted to ~37 ng µl^−1^ in a 50 µl volume following ONT recommendations [24]. Mechanical fragmentation using Covaris g-TUBEs^™^ was performed by centrifugation at 6,000 r.p.m. for two 60 s spins, targeting fragment sizes of ~10–15 kb to preserve long-read assembly contiguity while improving fragment length consistency for multiplexed sequencing. The resulting fragment distributions reached ~15 kb, within the expected range reported by a previous study that explored Covaris DNA fragmentation under comparable DNA input and centrifugal force conditions [23]. Fragment sizes were verified using Agilent TapeStation gDNA ScreenTape, and DNA concentrations were re-quantified using Qubit^™^ fluorometric assay before library preparation.

### Whole-genome sequencing

ONT sequencing was performed at the Department of Bacteriology, Institute of Tropical Medicine, Nagasaki University, Japan, using the Ligation Sequencing Kit with Native Barcoding 24 V14 (SQK-NBD114.24) and R10.4.1 flow cells (FLO-MIN114). All sequencing runs were performed using 400 ng of the input DNA sample, following the manufacturer’s protocol.

Two sequencing runs were conducted:

Run 1 – Controlled 6-plex benchmarking:

Unsheared and sheared DNA from Ty2, Ty42 and Ty43 were multiplexed (*n*=6) and sequenced for 72 h. Initial basecalling was performed in FAST (`dna_r10.4.1_e8.2_400bps_fast@v4.3.0`) mode. To standardize comparisons, all raw POD5 files were re-basecalled using Dorado v1.1.1. with:

- HAC (`dna_r10.4.1_e8.2_400bps_hac@v5.0.0`)
- SUP (`dna_r10.4.1_e8.2_400bps_sup@v5.0.0`)

Run 2 – High-throughput 24-plex field validation

Sheared DNA from 24 isolates (benchmark strains and 21 clinical isolates) was multiplexed and sequenced for 72 h using the same kit and flow cell type as Run 1. Basecalling was initially performed in SUP mode (model `dna_r10.4.1_e8.2_400bps_sup@v4.3.0`), and all raw data were subsequently re-basecalled using Dorado v1.1.1 with SUP mode (`dna_r10.4.1_e8.2_400bps_sup@v5.0.0`) to ensure consistency with Run 1 (Table 1).

**Table 1.** Study design and ONT workflows for Run 1 (controlled benchmarking) and Run 2 (field validation)

Run	Multiplex levels	Strains used	gDNA preparation	Initial basecaller mode	Final basecaller mode and model ID
**1**	6-plex	Ty2, Ty42, Ty43	Sheared	FAST@v4.3.0	HAC@v5.0.0; SUP@v5.0.0
**1**	6-plex	Ty2, Ty42, Ty43	Unsheared	FAST@v4.3.0	HAC@v5.0.0; SUP@v5.0.0
**2**	24-plex	Ty2, Ty42, Ty43 and 21 additional clinical isolates	Sheared	SUP@v4.3.0	SUP@v5.0.0

Run 1 (6-plex run) assessed DNA preparation (sheared vs unsheared) and basecalling mode (HAC vs SUP) on three benchmark *S*. Typhi strains. Run 2 (24-plex) applied the optimized workflow (sheared DNA+SUP) to 3 benchmark strains and 21 clinical isolates. All sequencing used the ONT Ligation Sequencing Kit with Native Barcoding 24 V14 (SQK-NBD114.24) and R10.4.1 flow cells. All datasets were initially basecalled with Dorado v0.7.2 and re-basecalled with Dorado v1.1.1 for final analyses.

HAC, high accuracy; SUP, super accuracy.

Demultiplexing was subsequently performed after basecalling using the ‘*dorado.exe demux*’ command with the ‘*--kit-name SQK-NBD114-24*’ and ‘*--emit-summary*’ flags to resolve individual barcoded isolates. Unless otherwise specified, all downstream benchmarking analyses were conducted using Dorado v1.1.1 with the HAC and SUP v5.0.0 model.

Short-read sequencing was performed at the NIID, Tokyo. gDNA was extracted using the MagMax DNA Multi-Sample Ultra 2.0 Kit with the KingFisher Duo Prime system (Thermo Fisher Scientific, USA). Libraries were prepared using the QIAseq FX DNA library kit (Qiagen) according to the manufacturer’s instructions and sequenced on an Illumina MiSeq instrument (2×300 bp paired-end reads).

### Genome assembly

#### ONT long-read assembly

Raw ONT reads were quality assessed with Nanoplot (v1.44.1) [32] and MultiQC (v1.28) [33]. Reads were then filtered with Filtlong (v0.2.1) [34] using the ‘*--min_length 1000 --keep_percent 95*’ options, following Hoffmann *et al*. [16]. Filtered reads were down-sampled to 100× coverage using Rasusa (v2.1.0) [35] to standardize coverage and comparisons across datasets.

Assemblies were generated using Flye (v2.9.5) [36] with the ‘*--nano-hq --read-error 0.03 --iterations 4 --genome-size 5.0 m*’ options, as previously described [16]. Assemblies were polished with three rounds of Medaka (v2.0.0) [37], using the corresponding HAC or SUP model.

#### Illumina short-read assembly

Raw Illumina reads were trimmed using fastp (v1.01) [38] and assembled *de novo* with SPAdes (v4.2.0) [39], followed by Pilon (v1.24) [40] polishing to correct small errors.

#### Hybrid assembly and reference genome selection

Strain-specific hybrid assemblies (Illumina short read+ONT long read) were generated using Hybracter (v0.11.0) [41], an automated long-read-first hybrid assembly workflow, using the ‘*hybrid-single*’ mode with ‘*--auto*’ and ‘*--no-medaka*’ options. For each strain (Ty2, Ty42 and Ty43), hybrid assemblies were constructed using the corresponding ONT sequencing datasets and Illumina MiSeq paired-end reads listed in Table S1.

To define ground truth references for benchmarking, hybrid assemblies generated from the 6-plex and 24-plex datasets were compared using MUMmer4 (v4.0.1) [42]. For each strain, the 24-plex sheared DNA reads (re-basecalled with SUP@v5.0.0) showed superior completeness and structural consistency required for high-quality reference genomes, including contiguity, structural integrity and base-level accuracy [13]. MUMmer4 alignments showed that Ty42 hybrids were identical at both SNV and structural levels, while Ty2 and Ty43 hybrids from the 24-plex run exhibited fewer breakpoints and improved structural continuity relative to 6-plex counterparts. These differences likely reflect run-specific variation in read distribution, fragment-size consistency and assembly stochasticity rather than a direct benefit of lower sequencing depth. Based on these comparisons, the 24-plex SUP-hybrid assemblies were selected as ground truth references for all downstream benchmarking analyses. MUMmer4 outputs are available as supporting files via Zenodo.

#### Assembly quality assessment

Assembly quality was evaluated using QUAST (v5.3.0) [43] via the GalaxyTrakr platform (v21.09) (https://galaxytrakr.org) [44]. Metrics included number of contigs, genome fraction (%), NGA50 (normalized genomic alignment N50, the length of the shortest contig needed to cover 50% of the reference after alignment), misassemblies (relocations, inversions and translocations) and error rate per 100 kb (sum of mismatches and indels per 100 kb). Comparisons were made between HAC, SUP and Illumina for both sheared and unsheared DNA, using hybrid assemblies as references.

### SNV analysis

#### SNV calling

Variants were identified using Snippy (v4.6.0) [45] with strain-specific hybrid assemblies as reference and default parameters. Outputs included core genome alignments, pairwise SNV distance matrices (snippy-core, snippy-dists) and mode-specific unique SNVs. Mode-specific unique SNVs were defined as SNVs present in one sequencing mode but absent from the other sequencing modes within the same strain after comparison against the corresponding strain-specific hybrid reference. Visualizations were generated in R (v4.5.1).

#### SNV-based cluster tree building

To assess clustering concordance across sequencing modes, a technical SNV-based phylogeny was constructed. This analysis was intended to evaluate strain-level placement and consistency across ONT, Illumina and hybrid assemblies, rather than to infer fine-scale transmission or evolutionary relationships. All assemblies were mapped to the public *S*. Typhi Ty2 reference genome (NC_004631.1) using Snippy (v4.6.0) to generate a unified core genome alignment (core.full.aln) [45]. Datasets included ONT SUP and HAC sheared assemblies from both 6-plex and 24-plex runs, strain-specific hybrid assemblies, Illumina assemblies, 19 SLH clinical isolates, 3 publicly available Philippine genomes (14ARS_GMH0152, 14ARS_VSM1035 and 14ARS_VSM1144) from BioProject PRJEB17615 (ENA), retrieved via the Pathogenwatch database (https://pathogen.watch) [46], and CT18 (AL513382.1). Recombination-associated regions were identified and masked using Gubbins (v3.0.0) [47] before phylogenetic inference. A maximum-likelihood (ML) phylogeny was then inferred from the recombination-filtered alignment using IQ-TREE (v3.0.1) under a GTR+G substitution model with 1,000 ultrafast bootstrap replicates [48, 49]. Trees were midpoint-rooted and visualized in iTOL (v7.2.2) [50], with additional annotation and formatting.

### cgMLST

Assemblies were uploaded to the Pathogenwatch platform (https://pathogen.watch), cgMLST v2, EnteroBase scheme (v1.2.0) for *S*. Typhi [46]. Allelic profiles were downloaded, and pairwise allelic distances were calculated and visualized as minimum spanning trees (MSTs) in R (v4.5.1), with hybrid assemblies used as the reference root. Pairwise cgMLST distances were defined as the number of loci with different allele calls between two assemblies. Loci with missing or absent allele calls in either assembly were excluded from pairwise distance calculation rather than counted as mismatches.

### Plasmid screening

To support the interpretation of assembly contiguity and secondary contigs, assemblies were screened for plasmid-associated sequences using MOB-suite (v3.1.9) [51, 52]. For the benchmark strains Ty2, Ty42 and Ty43, Illumina short-read assemblies, hybrid assemblies, SUP-sheared ONT-only assemblies and SUP-unsheared ONT-only assemblies were analysed. SUP-sheared assemblies from the 24-plex SLH field run were also screened descriptively. MOB-suite classifications were used to determine whether contigs were classified as plasmid-associated or chromosomal and whether plasmid-associated replicon or relaxase markers were detected. Because SLH field isolates were sequenced only using the sheared DNA workflow, they were not used to compare plasmid recovery between sheared and unsheared DNA preparations.

### Genotyping, serotyping, MLST and *in silico* AMR profiling

Genotyping and AMR profiling were performed using Mykrobe (v0.13.0) [53], directly from FASTQ files. Serotyping was conducted with SeqSero2 (v1.3.1) [54] via GalaxyTrakr using assembled genome FASTA files as input. SeqSero2 was run in k-mer mode with the genome assembly input setting (-m k -t 5) and default GalaxyTrakr parameters. A seven-gene MLST was assigned using the MLST tool (v2.22.0) [55, 56], also via the GalaxyTrakr [44].

### Phenotypic antimicrobial susceptibility testing

Phenotypic susceptibility testing was done using disc diffusion for first- and second-line *S*. Typhi antibiotics, including ampicillin, chloramphenicol, trimethoprim-sulfamethoxazole, cefixime (CFM), ceftriaxone, ciprofloxacin (CIP) and azithromycin.

Standardized antibiotic discs (BD BBL^™^ Sensi-Disc^™^, Becton, Dickinson and Company) were applied to LB agar plates inoculated with each isolate. After incubation at 37 °C for 18 h, the inhibition zone diameters were measured using the Colony Counter CC500 (v3.0.0.1), and the interpretations followed the CLSI M100-34^th^ edition (2024) guidelines [57].

### Computational environment

All downstream analyses were conducted with CPU-only computation on a Mac mini (2023, Apple M2 chip, 8-core CPU, 16 GB unified memory, 1 TB storage) and web-based platforms (GalaxyTrakr, Pathogenwatch).

Default parameters were used unless otherwise specified. A schematic overview of the analysis workflow is provided in Fig. S1.

## Results

### DNA quality and shearing efficiency

High-molecular-weight gDNA was obtained from three *S*. Typhi strains (Ty2, Ty42 and Ty43), with pre-shearing fragment sizes >48 kb. Mechanical shearing using Covaris g-TUBEs^™^ reduced fragment lengths to a modal distribution centred around ~15 kb (Fig. S2). DIN values remained high across samples (DIN 8.0–8.1), and all sheared profiles showed clean, high-integrity fragments suitable for long-read library preparation.

### Raw ONT read quality and yield across basecalling modes

To identify the optimal workflow, we first performed the controlled 6-plex run using the sheared and unsheared gDNA from three benchmark *S*. Typhi strains. Across the strains, SUP basecalling consistently produced higher read quality than HAC, increasing the mean Q score to ~Q18 range and ≥Q20 proportions from <5% to >75%. Read length distributions (mean read length and N50) were comparable (Table 2).

**Table 2.** Summarized metrics of raw ONT read quality in Run 1 (6-plex)

DNA preparation	Basecalling mode	Mean read length (kb)	Mean Q score	N50 (kb)	Total bases (Gbp)	>Q20 (%)	Pre-filtered coverage (×)
**Sheared**	HAC@5.0.0	7.87–8.91	14.3–14.5	9.41–10.56	6.01–7.61	2.8–3.1	1252–1584
**Sheared**	SUP@5.0.0	7.91–8.95	17.9–18.1	9.44–10.59	6.16–7.79	74.2–75.4	1283–1623
**Unsheared**	HAC@5.0.0	6.61–8.25	14.4–14.5	10.09–13.82	0.35–1.12	4.1–4.7	73–319
**Unsheared**	SUP@5.0.0	6.68–8.32	17.9–18.1	10.28–13.95	0.36–1.16	72.7–73.7	76–330

Values represent ranges across three benchmark strains (Ty2, Ty42 and Ty43) under sheared and unsheared DNA preparations with HAC or SUP basecalling. Metrics were generated using NanoPlot and summarized with MultiQC. Coverage was estimated relative to a 4.8 Mb genome size prior to filtering.

HAC, high accuracy basecalling; N50, read length at which 50% of total bases are contained in reads of that length or longer; SUP, super accuracy basecalling.

DNA preparation had a marked effect on sequencing yield. Before read filtering, sheared DNA yielded >6 Gbp per strain (1,252×–1,623× coverage), whereas unsheared DNA showed markedly reduced throughput (0.35–1.16 Gbp; 73×–330× coverage) (Table 2). After read filtering and Rasusa downsampling to ~100× coverage, all sheared datasets achieved the target depth, while among unsheared datasets, Ty43 did not meet the threshold, achieving ~68× coverage with HAC basecalling and ~70× coverage with SUP basecalling (Table S2).

### Assembly completeness and structural metrics compared to hybrid references

After *de novo* assembly with Flye and three rounds of Medaka polishing, assembly contiguity was broadly comparable between sheared and unsheared DNA preparations, although strain- and mode-specific differences were observed (Table S2). MOB-suite screening did not classify any benchmark assembly contigs as plasmids, indicating that observed contig-number differences primarily reflected chromosomal assembly fragmentation rather than plasmid reconstruction (Table S5).

**Fig. 2.**
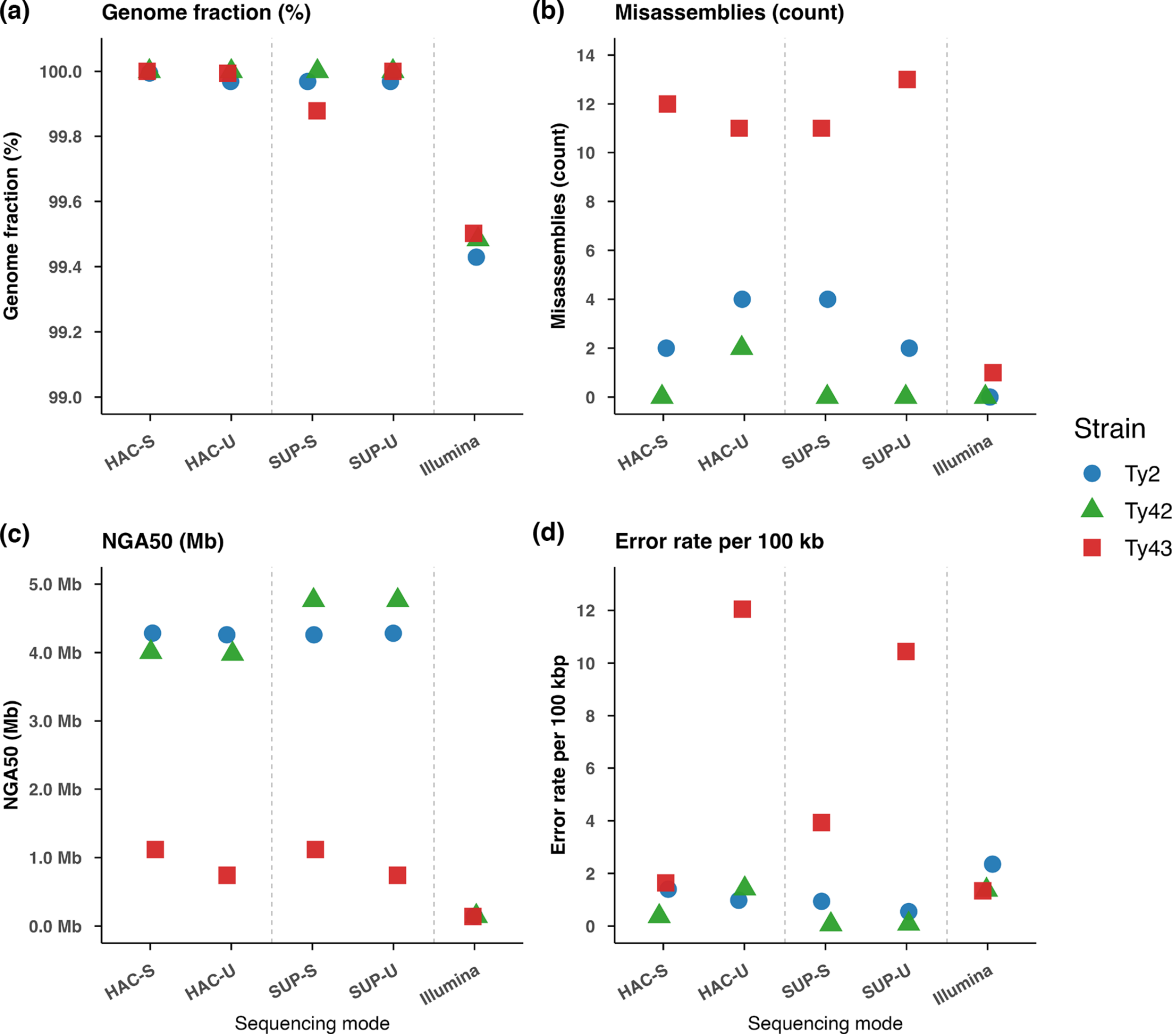
Strain-level assembly metrics across sequencing modes. QUAST metrics comparing ONT HAC, ONT SUP and Illumina assemblies against strain-specific hybrid references (approx. length 4.69–4.77 Mb) for Ty2, Ty42 and Ty43. Each point represents one benchmark strain. Vertical dashed lines separate HAC, SUP and Illumina sequencing conditions. Panels show (**a**) genome fraction (%), (**b**) misassemblies (count), (**c**) NGA50 (Mb) and (**d**) error rate per 100 kb. Genome fraction, proportion of the reference genome recovered; NGA50, aligned contig length at which 50% of the reference genome is covered; S, sheared DNA; U, unsheared DNA; HAC, high accuracy basecalling; SUP, super accuracy basecalling; misassemblies, structural errors such as inversions, relocations or translocations; error rate per 100 kb, base-level mismatches+indels per 100,000 aligned bases.

As each sequencing condition included three benchmark strains, QUAST metrics are shown as individual strain-level values using dot plots (Fig. 2). Genome fraction was consistently high across ONT assemblies, exceeding 99.9% of their corresponding hybrid references (strain-specific assemblies generated from combined ONT long reads and Illumina short reads), while Illumina showed the expected slightly lower breadth (99.4–99.5%) (Fig. 2a). Misassembly counts varied mainly by strain rather than DNA preparation alone, with Ty43 showing higher counts across several ONT conditions (Fig. 2b). NGA50 values were highest in SUP assemblies (>4 Mb) and consistently lower in HAC, with Illumina approaching zero due to short-read assembly fragmentation (Fig. 2c). Error-rate profiles (mismatches+indels per 100 kb) were generally lower for Ty2 and Ty42 and higher for Ty43, especially in unsheared HAC and SUP datasets (Fig. 2d).

Overall, mechanically sheared DNA showed assembly metrics comparable to unsheared DNA across accuracy metrics, while demonstrating reduced variability in structural error profiles under multiplexed conditions. While strain-specific differences were evident, particularly for Ty43, mechanical DNA shearing contributed to more consistent assembly performance across sequencing conditions without impacting assembly accuracy.

### SNV concordance and mode-specific artefacts across DNA types and basecalling modes

**Fig. 3.**
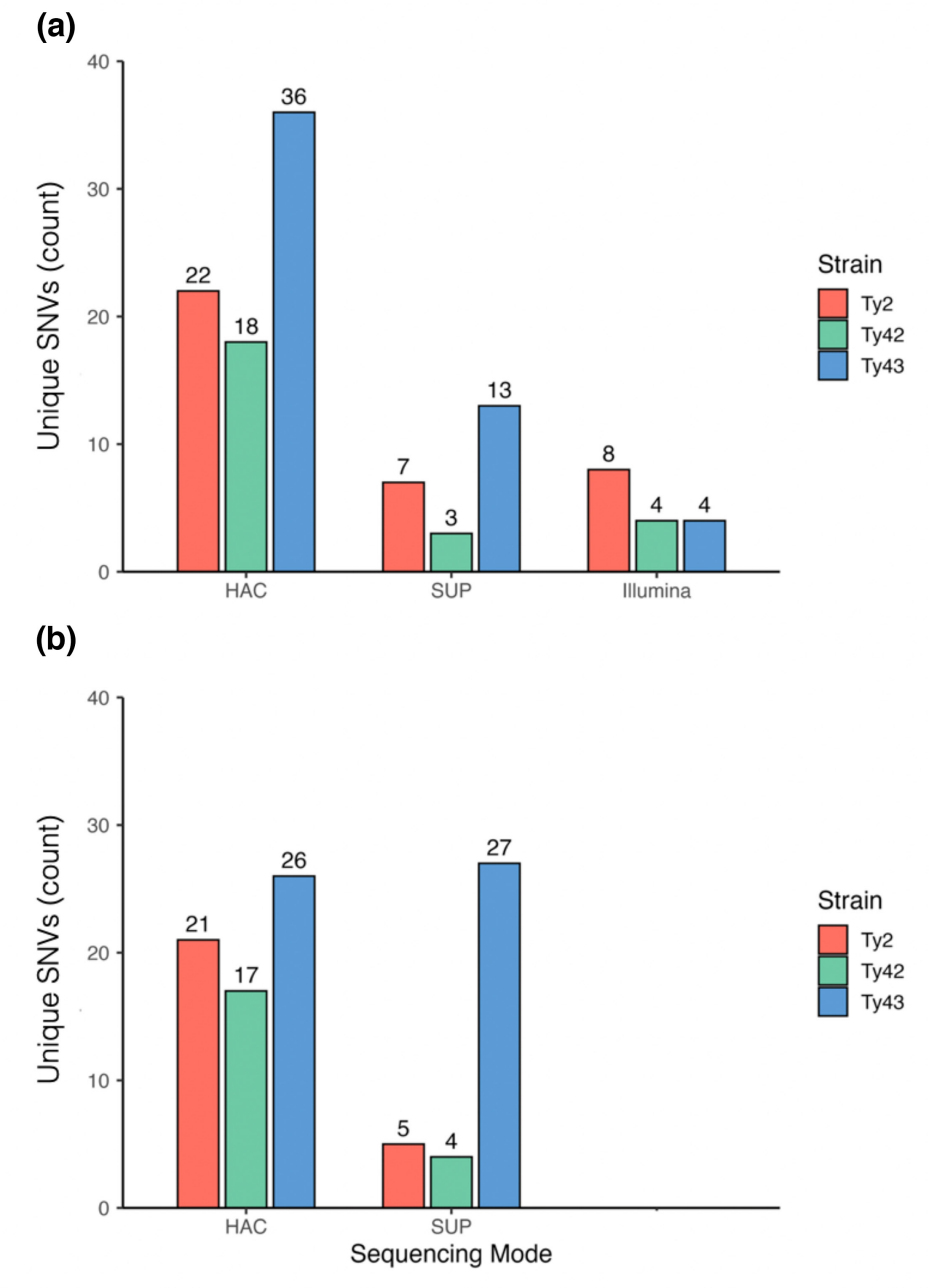
Mode-specific SNV differences across sequencing conditions. Panels show SNVs detected uniquely in different sequencing modes for (**a**) sheared DNA and Illumina assemblies and (**b**) unsheared DNA assemblies. SNVs were identified relative to the corresponding strain-specific hybrid references. Bars indicate the number of unique SNVs present in one sequencing mode but absent from all other modes within the same strain. Numbers above bars indicate the exact unique SNV counts. These unique SNVs represent mode-specific variation relative to the hybrid references. HAC, high accuracy basecalling; SUP, super accuracy basecalling.

SNVs provide the highest phylogenetic resolution for a genetically monomorphic pathogen such as *S*. Typhi and are essential for distinguishing circulating lineages in modern typhoid surveillance systems [4, 58]. Across the sequencing modes, the basecalling strategy had the strongest influence on mode-specific SNV differences (Fig. 3). In sheared DNA samples, SUP basecalling consistently produced the fewest mode-specific SNVs (3–13 per strain) overlapping with the Illumina range [4–8], whereas HAC introduced much higher counts (18–36 per strain) (Fig. 3a).

The effect of DNA shearing on SNV artefacts was modest and basecalling-dependent. Under HAC, unsheared libraries showed slightly lower SNV counts than sheared libraries, whereas under SUP, the differences were most pronounced in Ty43 (13 vs 27 unique SNVs in sheared vs unsheared, respectively) (Fig. 3b). Overall, sheared datasets performed similarly to unsheared DNA at the SNV level and did not cause substantial inflation of artefacts.

Pairwise SNV distances further supported these findings. For Ty2 and Ty42, ONT assemblies differed from their corresponding hybrid references by 0–4 SNVs across sequencing conditions, whereas larger distances were observed for Ty43 (3–16 SNVs), particularly in the SUP-unsheared dataset (Fig. S3A–C). The consistently higher SNV distances observed for Ty43, despite identical laboratory processing and analytical workflows, suggest that residual ONT discordance may also be influenced by strain-dependent factors in addition to DNA preparation or basecalling mode.

To assess whether these differences affected phylogenetic placement, a recombination-filtered ML phylogeny was reconstructed from core SNVs generated across the 6-plex and 24-plex sheared ONT datasets using the public Ty2 reference genome (NC_004631.1). Gubbins identified 49 predicted recombination-associated regions within the core-SNP alignment. In a highly clonal pathogen such as *S*. Typhi, these regions may also represent clustered variation associated with prophage, repetitive or other problematic genomic regions. Predicted regions were identified with Gubbins and masked before phylogenetic inference. Despite the pairwise SNV differences observed between assemblies (Fig. S3), benchmark assemblies from Ty2, Ty42 and Ty43 consistently clustered with their corresponding hybrid and Illumina references. This analysis was intended to assess strain-level clustering and broader phylogenetic contextualization rather than fine-scale outbreak reconstruction, as residual SNV discordance remained among technical assemblies (Fig. S5).

### cgMLST allele concordance across basecalling modes

**Fig. 4.**
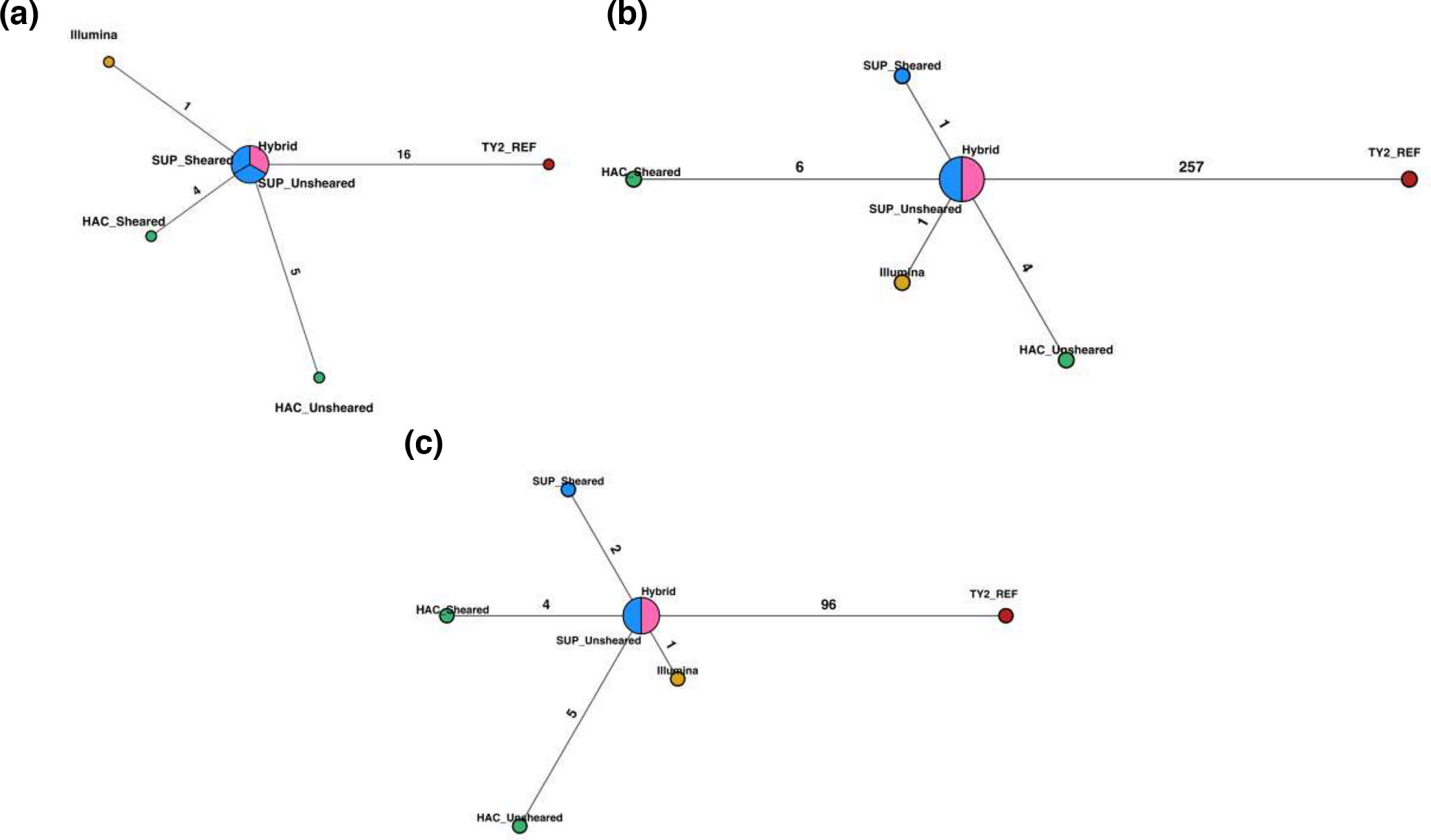
cgMLST MSTs for genome assemblies across different DNA preparation types and sequencing modes. MSTs showing cgMLST allele differences among assemblies generated from different DNA preparations and sequencing modes for (**a**) Ty2, (**b**) Ty42 and (**c**) Ty43. Trees were constructed using 3,002 shared core-genome loci. Numbers on edges indicate pairwise cgMLST allele differences between connected nodes. Hybrid assemblies were used as strain-specific references for comparison. HAC, high accuracy basecalling; SUP, super accuracy basecalling; TY2_REF, Ty2 reference genome NC_004631.1.

To complement SNV analysis and evaluate suitability for routine public health surveillance, cgMLST profiling was performed using the EnteroBase *S*. Typhi scheme via the Pathogenwatch database. National and regional reference laboratories widely adopt allele-based approaches because they provide stable clustering across sequencing platforms and analytical pipelines and are less sensitive to minor methodological variation [59]. Across all benchmark *S*. Typhi strains, SUP assemblies showed near-identical allele profiles relative to hybrid references, clustering within zero to two alleles (Fig. 4a–c). Conversely, HAC assemblies deviated more (typically four to six alleles), especially for Ty43. The DNA preparation method (sheared vs unsheared) had minimal impact on cgMLST concordance, with both preparations clustering closely with their respective hybrid references (Fig. 4).

### Genotyping, serotyping, MLST and AMR profiling

Genotype, serotype and MLST predictions showed complete concordance across all ONT (HAC/SUP) and Illumina assemblies. No known AMR determinants were detected by Mykrobe in all benchmark strains (Table 3). Phenotypic susceptibility testing showed that Ty2 was susceptible to all antibiotics tested, whereas Ty42 demonstrated intermediate susceptibility to CFM and Ty43 demonstrated intermediate susceptibility to CIP. Despite these phenotypic differences, genomic predictions remained concordant across all sequencing conditions and analytical workflows (Table 3).

**Table 3.** Genotyping, serotyping, MLST, *in silico* AMR predictions and phenotypic antibiotic susceptibility profiles for Run 1 (6-plex)

Strain	ST	Serotype	Genotype	Antigenic profile	O antigen	H1 antigen	*In silico* Mykrobe AMR predictions	Disc diffusion (mm) AMP/CHL/SXT/CFM/CRO/CIP/AZM	Interpretations (CLSI-M100 34th)
**Ty2**	1	Typhi	4.1	9: d:-	9	d	No known AMR determinants detected	30.83/26.67/28.49/33.55/30.83/26.67/28.49	All S
**Ty42**	2	Typhi	2.3.3	9: d:-	9	d	No known AMR determinants detected	26.97/24.83/25.48/20.79* /26.97/24.83/25.48	S except CFM=I
**Ty43**	1	Typhi	4	9: j:-	9	j	No known AMR determinants detected	24.61/25.03/24.83/26.27/30.27/29.42*/19.12	S except CIP=I

Genotype (Mykrobe), serotype (SeqSero2), MLST and *in silico *AMR predictions for benchmark strains across ONT (HAC, SUP), Illumina and hybrid assemblies. Phenotypic susceptibility was determined by disc diffusion and interpreted according to CLSI M100 (34th edition).

AMP, ampicillin; AZM, azithromycin; CFM, cefixime; CHL, chloramphenicol; CIP, ciprofloxacin; CRO, ceftriaxone; I, intermediate; S, susceptible; SXT, trimethoprim–sulfamethoxazole.

### Performance of the sheared DNA workflow in 24-plex multiplexing

The 6-plex benchmarking analysis (Run 1) demonstrated that mechanically sheared DNA paired with SUP basecalling at ~100× depth yielded the most stable ONT assemblies, across structural and SNV metrics. On this basis, the sheared DNA+SUP workflow was selected for evaluation under high-multiplex conditions in a 24-plex run (Run 2).

**Fig. 5.**
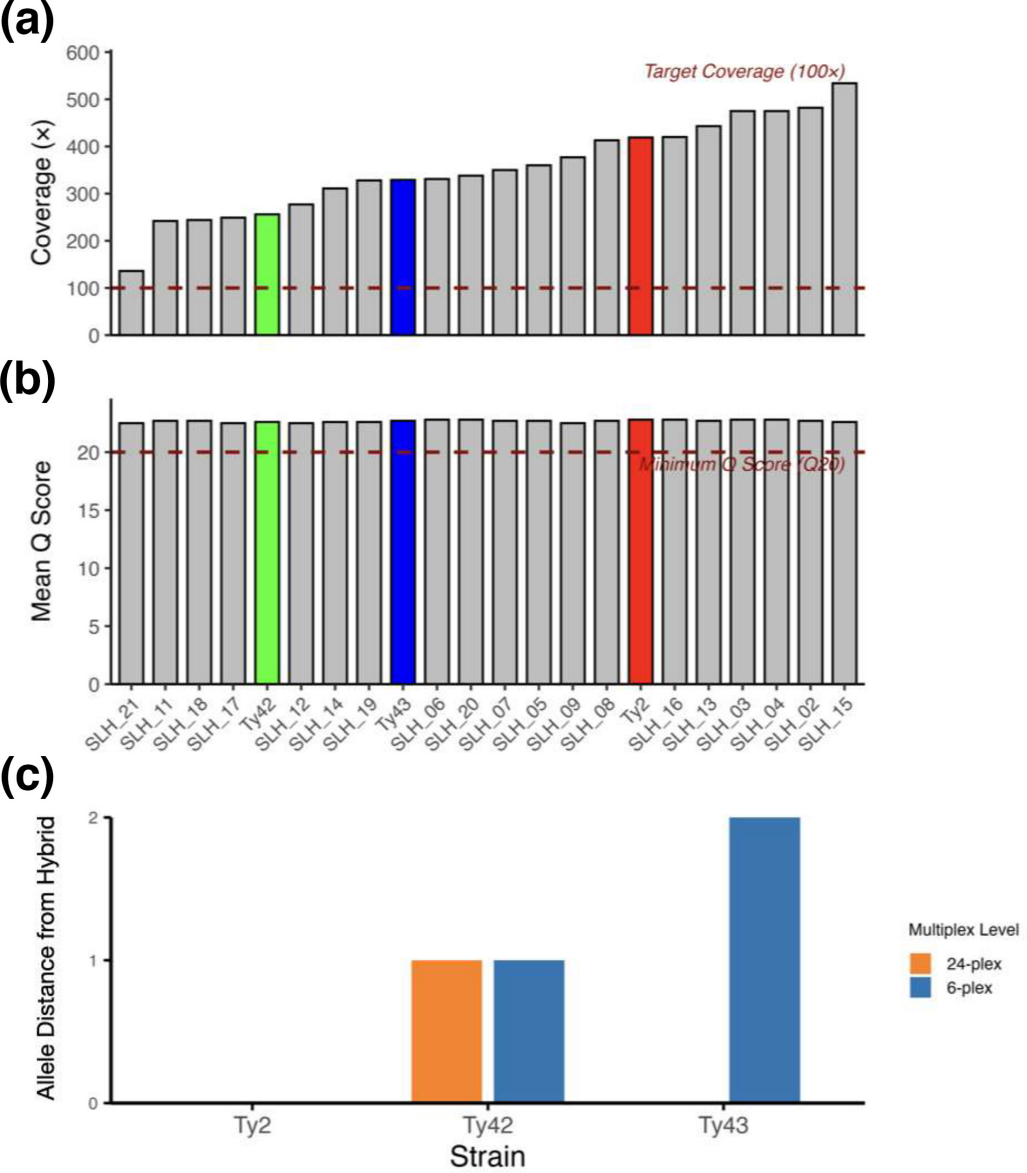
24-plex field run sequencing performance and cgMLST concordance. Panels: (**a**) Per-sample sequencing depth (× coverage); dashed red line indicates the target depth of 100×. (**b**) Mean per-read Q scores for the same samples; dashed red line indicates Q20. (**c**) cgMLST allele distances between 6-plex and 24-plex SUP assemblies for the three benchmark strains (Ty2, Ty42 and Ty43), relative to hybrid references. Benchmark strains are highlighted (Ty2, red; Ty42, green; Ty43, blue).

In Run 2, all samples achieved >100× coverage pre-filtering and ~100× coverage following downsampling with mean per-read Q scores>22 (Fig. 5a, b). Among the 24 samples, including 3 benchmark strains and 21 SLH clinical isolates, 18 assemblies were fully circularized. After excluding two isolates that were not confirmed as *S*. Typhi, 18 of the 22 retained *S*. Typhi genomes (81.8%) yielded fully circularized assemblies (Table S3). All three benchmark strains showed complete concordance in genotyping, serotyping and *in silico* AMR prediction relative to Run 1, with no known AMR determinants detected. Two non-Typhi isolates were excluded from further analyses (Table S4). Descriptive MOB-suite screening of the SLH field-run assemblies did not classify any contigs as plasmids; however, SLH_04 contained plasmid-associated replicon and relaxase markers on contigs classified as chromosomal, and these were not interpreted as reconstructed plasmids (Table S5).

To assess reproducibility at higher multiplexity, benchmark assemblies from Run 2 were compared with their SUP-sheared counterparts from Run 1. QUAST metrics showed comparable genome fractions and structural performance across runs. Misassembly and error rate per 100 kb varied by strain but remained within the same overall range, indicating that increased multiplexing did not substantially compromise structural fidelity (Table S6). cgMLST concordance remained high across runs, with allelic distances ranging from 0 to 2 alleles relative to hybrid references (Fig. 5c), confirming stable allele-level clustering under high-multiplex conditions. Pairwise SNV distances were generally comparable to or lower than Run 1, except for Ty2, which reached 19 SNVs (Fig. S4). This increase, in the absence of cgMLST or assembly metrics changes, likely reflects residual base-level consensus shifts introduced during rebasecalling rather than true genomic divergence.

In the same recombination-filtered ML phylogeny, SLH_09 was excluded from the displayed tree as it had an identical filtered core-SNP profile to SLH_05. These isolates had different sample identifiers, and available sample metadata did not indicate duplicate sampling. The remaining 18 SLH clinical isolates clustered according to their Mykrobe-assigned genotypes (e.g. 3, 3.2.1 and 4.1) and grouped consistently with the 3 publicly available Philippine genomes (14ARS collection) included for comparison (Fig. S5). Although the Ty2 assembly exhibited a slightly longer branch than its 6-plex counterparts, it remained nested within the Ty2 clade with strong bootstrap support, indicating a preserved phylogenetic placement. Overall, these results demonstrated that the sheared DNA+SUP basecalling workflow preserves assembly integrity, functional reliability and strain-level phylogenetic structure under 24-plex multiplexed conditions.

## Discussion

In this study, we evaluated whether mechanically sheared DNA, combined with HAC or SUP basecalling, supports ONT-based *S*. Typhi genomic surveillance in a multiplexed sentinel hospital setting. Through controlled 6-plex benchmarking and a 24-plex field run evaluation, we found that sheared DNA combined with SUP basecalling at ~100× depth produced assemblies comparable to Illumina and hybrid references for SNV- and cgMLST-based analyses, while maintaining concordant genotyping, serotype prediction, MLST assignment and *in silico* AMR outputs across benchmark strains. As this evaluation was based on two sequencing runs, the findings should be interpreted as proof-of-concept evidence requiring further replication across additional flow cells, library preparations and laboratory settings.

Long-read sequencing workflows often prioritize preservation of high-molecular-weight DNA to maximize read length, which can help resolve repetitive bacterial genomes. However, this approach does not necessarily ensure balanced per-sample coverage in high multiplex runs [18, 19, 60]. In sentinel laboratories, where multiple isolates may be batched on a single flow cell to reduce cost, uneven read distribution can compromise downstream analyses if some samples fail to reach minimum depth thresholds [6, 18]. In our 6-plex benchmarking run, unsheared libraries showed greater variability in pre- and post-filtered coverage, and some failed to consistently reach the 100× target used for standardized benchmarking of SNV and cgMLST concordance. In contrast, mechanically sheared libraries achieved consistent depth (>1,000×) across all samples. Importantly, DNA shearing did not introduce systematic degradation of assembly quality. Across structural, SNV and allele-based metrics, sheared assemblies performed comparably to unsheared datasets. These findings suggest that controlled input-DNA fragmentation can improve coverage stability in multiplexed ONT workflows without compromising routine phylogenetic or functional outputs.

Previous ONT multiplexing studies for *Salmonella* serotyping have identified uneven read distribution as a constraint and suggested limiting isolates per flow cell, typically to five, to maintain adequate per-sample depth for rapid serotype classification [17, 20]. These studies, together with related ONT evaluations, have shown that lower coverage (~30×−50×) may support targeted surveillance outputs such as serotype prediction and detection of known genetic markers [17, 20, 21]. Therefore, the 100× coverage target used here should be interpreted as a conservative standardized threshold for comparing assembly performance, SNV concordance, cgMLST profiling and phylogenetic contextualization across sequencing conditions, not as a universal requirement for all ONT-based surveillance applications. In resource-constrained sentinel settings, higher multiplexing remains a trade-off between cost, throughput, turnaround time and depth-dependent genomic resolution [13, 61].

Basecalling mode had a greater effect than DNA preparation on SNV concordance and cgMLST stability. SUP mode consistently produced more stable results than HAC, particularly at the SNV level, reinforcing that basecaller selection remains central to phylogenetic reliability in ONT-based workflows [16]. We also observed consistent strain-specific differences in assembly performance, particularly for benchmark strain Ty43, which retained higher SNV counts relative to its hybrid reference despite identical laboratory and bioinformatic processing. Similar strain-dependent variability has been reported in large-scale evaluations of ONT-only sequencing, where a small subset of bacterial isolates exhibited elevated consensus error rates associated with lineage-specific DNA modification systems and other intrinsic genomic characteristics [28]. These findings suggest that ONT assembly accuracy is influenced not only by sequencing depth and basecalling model but also by biological features of individual genomes that affect consensus generation. Although methylation or other DNA modification patterns were not examined in the present study, such factors may have contributed to the elevated SNV burden observed in Ty43.

After masking predicted recombination-associated regions, benchmark assemblies clustered with their corresponding Illumina and hybrid references in the technical phylogeny, supporting stable strain-level placement across sequencing approaches. However, residual SNV differences remained between ONT-only and reference assemblies. This is particularly relevant for outbreak investigations, where epidemiologically linked *Salmonella* isolates may differ by only a small number of core SNPs [62]. In such cases, consensus-level ONT errors of similar magnitude could potentially influence the interpretation of transmission links and outbreak boundaries. Therefore, while the sheared DNA SUP workflow appears sufficient for routine surveillance lineage assignment and phylogenetic contextualization, short-read or hybrid sequencing remains preferable when maximum SNP-level resolution is required for fine-scale transmission analysis [16, 61, 62]. Plasmid reconstruction is also an important advantage of long-read sequencing for bacterial genomic surveillance, particularly because plasmids can carry AMR determinants in *Salmonella* and may contain repetitive regions that are difficult to resolve with short reads alone [63]. In this study, MOB-suite did not classify any benchmark or SLH field-run contigs as plasmids. Therefore, the effect of ~15 kb mechanical shearing on plasmid recovery could not be directly assessed. Similarly, *in silico* AMR outputs were concordant across sequencing conditions, but the three benchmark strains lacked acquired AMR genes and known resistance-associated mutations. Broader validation across resistant, plasmid-bearing and genetically diverse *S*. Typhi lineages will therefore be required.

The workflow evaluated here used ligation-based native barcoding, and the findings should be interpreted within this library preparation context. Other ONT library strategies, including rapid transposase-based and PCR-based barcoding approaches, may produce different coverage distributions and could potentially improve multiplexing performance in some settings [64]. However, these approaches also differ in read-length distribution, DNA input requirements, amplification bias, hands-on time and suitability for assembly- and consensus-based analyses. Future studies should directly compare mechanical shearing with alternative ONT library preparation strategies for high-multiplex bacterial genomic surveillance. Nonetheless, overall findings from this study support mechanical shearing as a simple and practical modification for multiplexed ONT workflows that can improve coverage stability without compromising contextual phylogenetic placement or functional reliability.

Strengthening early pathogen detection and genomic surveillance in frontline laboratory settings is a key priority under the 2025 WHO Pandemic Agreement and global genomic frameworks [11, 65]. Sentinel hospitals serve as the primary interface between emerging infections and public health response, yet many still rely on centralized reference facilities, leading to delays and limited local analytical capacity [6, 7]. This study supports the feasibility of high-multiplex ONT sequencing in such settings, but generating sequence data locally does not by itself eliminate barriers to genomic surveillance implementation. While web-accessible platforms such as GalaxyTrakr [44] and Pathogenwatch [46] can support parts of the analytical workflow and reduce the complexity of bioinformatics infrastructure, key outputs, including AMR prediction and customized analyses, may still require command-line tools, local installation and experienced bioinformatics support. Workflow cost is also an important consideration. Covaris g-TUBEs^™^ provided a reproducible approach for generating ~15 kb fragments using only a standard benchtop centrifuge rather than dedicated shearing equipment, but they add consumable cost and may not be the most affordable option for all sentinel laboratories [23, 24]. Alternative fragmentation or library preparation approaches, including needle shearing, enzymatic fragmentation, sonication, bead- or vortex-assisted shearing and rapid transposase-based workflows, may be more accessible in some settings but require further validation because they differ in reproducibility, DNA input requirements, fragment-size control, read-length distribution, assembly contiguity and consensus accuracy [66–68]. Therefore, Covaris g-TUBEs^™^ shearing should be viewed as a reproducible proof-of-concept strategy that trades higher consumable cost for operational simplicity and fragment-size consistency, rather than as the only implementable fragmentation method. A practical implementation model may involve sentinel hospitals generating high-quality sequence data locally, while national or regional reference laboratories support quality assurance, pipeline standardization and integration into broader surveillance systems [11, 69].

This study has the following limitations. First, benchmarking was performed on three *S*. Typhi benchmark strains that lacked acquired AMR genes, known resistance-associated mutations and MOB-suite-detected plasmids. Although *in silico* AMR outputs were concordant across sequencing conditions, this study could not directly evaluate resistance lineages, plasmid-borne AMR genes or the effect of mechanical shearing on plasmid recovery. Previous ONT benchmarking studies have reported high concordance for AMR gene detection when adequate read quality and coverage are achieved [21, 53], but broader validation across resistant, plasmid-bearing and genetically diverse *S*. Typhi lineages remains necessary. Second, only one mechanical shearing target (~15 kb) was evaluated; alternative fragment sizes or library preparation strategies may perform differently and should be evaluated in future studies. Third, we tested specific ONT chemistries and basecalling models (HAC v5.0.0 and SUP v5.0.0 with Dorado v1.1.1), and ongoing changes to ONT platforms may alter performance characteristics. Fourth, the study evaluated one controlled 6-plex benchmarking run and one 24-plex field run. Because ONT sequencing performance can vary between flow cells, library preparations and sequencing runs, additional technical replicates and independent evaluations across laboratories will be required to determine the reproducibility and generalizability of the observed benefits of mechanical DNA shearing. Finally, although recombination-associated regions were masked before phylogenetic reconstruction, SNP analyses were performed on consensus assemblies rather than read-level variant calls. Future studies incorporating read-level variant calling and masking of ambiguous positions may further improve resolution for fine-scale outbreak investigations.

## Conclusion

As sequencing technologies advance, the challenge in public health genomics is shifting from data generation to reliable implementation in frontline settings. This study demonstrates that modest workflow adaptations, such as mechanical DNA shearing combined with SUP basecalling mode, provide the consistency required for routine genomic surveillance without increasing analytical complexity. While short-read or hybrid sequencing remains important for high-resolution transmission analysis, ONT-only workflows optimized for coverage stability offer a practical, viable path for decentralized surveillance when supported by appropriate quality assurance, bioinformatics support and partnerships with reference laboratories. Future work should extend this approach to more diverse, resistant and plasmid-bearing *S*. Typhi populations; evaluate low-cost fragmentation strategies; and assess how ongoing improvements in ONT chemistry and basecalling further strengthen its role in public health genomics.

## Supplementary material

10.1099/mgen.0.001805 Supplementary Material 1.

## Abbreviations

AMR: antimicrobial resistance
CFM: cefixime
cgMLST: core-genome MLST
CIP: ciprofloxacin
DIN: DNA Integrity Number
gDNA: genomic DNA
LB: Luria–Bertani
LMICs: low- and middle-income countries
ML: maximum-likelihood
MSTs: minimum spanning trees
NGA50: normalized genomic alignment N50
NIID: National Institute of Infectious Diseases
ONT: Oxford Nanopore Technologies
SLH: San Lazaro Hospital
SNV: single-nucleotide variation
*S*. Typhi: *Salmonella enterica* serovar Typhi
SUP: super accuracy
