# Supplementary material for: Performance of mechanically sheared DNA in multiplexed Oxford Nanopore sequencing for Salmonella Typhi genomic surveillance
Source: Microb Genom. 2026 Jul 27;12(7):001805. doi: 10.1099/mgen.0.001805 (PMC13405487; doi:10.1099/mgen.0.001805)
Supplement: Supplementary Material 1. [file mgen-12-01805-s001.pdf]

Supplementary Materials

Supplementary Table S1. Annotated assembly and raw sequence accession numbers for all bacterial isolates' sequence data included in the study

Organism	Assigned name	Sample type	Source	Sequencing dataset	BioSample accessions	Annotated assembly accessions	DRA raw sequence accessions
***Salmonella enterica* subsp. *enterica* Typhi**	Ty2	Reference strain	NIID, Tokyo	ONT SUP sheared 6-plex	SAMD01795632	BAAJZN010000001-BAAJZN010000002	DRR911410
				ONT SUP unsheared 6-plex		BAAJZO010000001-BAAJZO010000002	DRR911435
				ONT HAC sheared 6-plex		BAAJZK010000001-BAAJZK010000002	DRR911438
				ONT HAC unsheared 6-plex		BAAJZJ010000001-BAAJZJ010000002	DRR911441
				ONT SUP sheared 24-plex		BAAJZI010000001-BAAJZI010000002	DRR911444
				Illumina MiSeq (2 × 300bp)		BAAJZM010000001-BAAJZM010000086	DRR911432
				Hybrid assembly		BAAJZL010000001-BAAJZL010000002	-
***Salmonella enterica* subsp. *enterica* Typhi**	Ty42	Clinical isolate	NIID, Tokyo	ONT SUP sheared 6-plex	SAMD01795633	BAAJZU010000001	DRR911411
				ONT SUP unsheared 6-plex		BAAJZV010000001	DRR911436
				ONT HAC sheared 6-plex		BAAJZQ010000001-BAAJZQ010000003	DRR911439
				ONT HAC unsheared 6-plex		BAAJZR010000001-BAAJZR010000005	DRR911442
				ONT SUP sheared 24-plex		BAAJZP010000001	DRR911445
				Illumina MiSeq (2 × 300bp)		BAAJZT010000001-BAAJZT010000094	DRR911433
				Hybrid assembly		BAAJZS010000001	-
***Salmonella enterica* subsp. *enterica* Typhi**	Ty43	Clinical isolate	NIID, Tokyo	ONT SUP sheared 6-plex	SAMD01795634	BAAKAB010000001-BAAKAB010000006	DRR911412
				ONT SUP unsheared 6-plex		BAAKAC010000001-BAAKAC010000006	DRR911437
				ONT HAC sheared 6-plex		BAAJZX010000001	DRR911440
				ONT HAC unsheared 6-plex		BAAJZY010000001-BAAJZY010000006	DRR911443

				ONT SUP sheared 24-plex		BAAJZW010000001-BAAJZW010000007	DRR911446
				Illumina MiSeq (2 × 300bp)		BAAKAA010000001-BAAKAA010000118	DRR911434
				Hybrid assembly		BAAJZZ010000001-BAAJZZ010000007	-
***Salmonella enterica* subsp. *enterica* Typhi**	SLH_02	Clinical isolate	SLH, Manila (CAB Study)	ONT SUP sheared 24-plex	SAMD01795635	BAAKAD010000001	DRR911413
***Salmonella enterica* subsp. *enterica* Typhi**	SLH_03	Clinical isolate	SLH, Manila (CAB Study)	ONT SUP sheared 24-plex	SAMD01795636	BAAKAE010000001	DRR911414
***Salmonella enterica* subsp. *enterica* Typhi**	SLH_04	Clinical isolate	SLH, Manila (CAB Study)	ONT SUP sheared 24-plex	SAMD01795637	BAAKAF010000001-BAAKAF010000005	DRR911415
***Salmonella enterica* subsp. *enterica* Typhi**	SLH_05	Clinical isolate	SLH, Manila (CAB Study)	ONT SUP sheared 24-plex	SAMD01795638	BAAKAG010000001	DRR911416
***Salmonella enterica* subsp. *enterica* Typhi**	SLH_06	Clinical isolate	SLH, Manila (CAB Study)	ONT SUP sheared 24-plex	SAMD01795639	BAAKAH010000001	DRR911417
***Salmonella enterica* subsp. *enterica* Typhi**	SLH_07	Clinical isolate	SLH, Manila (CAB Study)	ONT SUP sheared 24-plex	SAMD01795640	BAAKAI010000001	DRR911418
***Salmonella enterica* subsp. *enterica* Typhi**	SLH_08	Clinical isolate	SLH, Manila (CAB Study)	ONT SUP sheared 24-plex	SAMD01795641	BAAKAJ010000001	DRR911419
***Salmonella enterica* subsp. *enterica* Typhi**	SLH_09	Clinical isolate	SLH, Manila (CAB Study)	ONT SUP sheared 24-plex	SAMD01795642	BAAKAK010000001	DRR911420
***Salmonella enterica* subsp. *enterica* Typhi**	SLH_11	Clinical isolate	SLH, Manila (CAB Study)	ONT SUP sheared 24-plex	SAMD01795643	BAAKAL010000001	DRR911421
***Salmonella enterica* subsp. *enterica* Typhi**	SLH_12	Clinical isolate	SLH, Manila (CAB Study)	ONT SUP sheared 24-plex	SAMD01795644	BAAKAM010000001-BAAKAM010000004	DRR911422

***Salmonella enterica* subsp. *enterica* Typhi**	SLH_13	Clinical isolate	SLH, Manila (CAB Study)	ONT SUP sheared 24-plex	SAMD01795645	BAAKAN010000001	DRR911423
***Salmonella enterica* subsp. *enterica* Typhi**	SLH_14	Clinical isolate	SLH, Manila (CAB Study)	ONT SUP sheared 24-plex	SAMD01795646	BAAKAO010000001	DRR911424
***Salmonella enterica* subsp. *enterica* Typhi**	SLH_15	Clinical isolate	SLH, Manila (CAB Study)	ONT SUP sheared 24-plex	SAMD01795647	BAAKAP010000001	DRR911425
***Salmonella enterica* subsp. *enterica* Typhi**	SLH_16	Clinical isolate	SLH, Manila (CAB Study)	ONT SUP sheared 24-plex	SAMD01795648	BAAKAQ010000001	DRR911426
***Salmonella enterica* subsp. *enterica* Typhi**	SLH_17	Clinical isolate	SLH, Manila (CAB Study)	ONT SUP sheared 24-plex	SAMD01795649	BAAKAR010000001	DRR911427
***Salmonella enterica* subsp. *enterica* Typhi**	SLH_18	Clinical isolate	SLH, Manila (CAB Study)	ONT SUP sheared 24-plex	SAMD01795650	BAAKAS010000001	DRR911428
***Salmonella enterica* subsp. *enterica* Typhi**	SLH_19	Clinical isolate	SLH, Manila (CAB Study)	ONT SUP sheared 24-plex	SAMD01795651	BAAKAT010000001	DRR911429
***Salmonella enterica* subsp. *enterica* Typhi**	SLH_20	Clinical isolate	SLH, Manila (CAB Study)	ONT SUP sheared 24-plex	SAMD01795652	BAAKAU010000001	DRR911430
***Salmonella enterica* subsp. *enterica* Typhi**	SLH_21	Clinical isolate	SLH, Manila (CAB Study)	ONT SUP sheared 24-plex	SAMD01795653	BAAKAV010000001	DRR911431
**Non-Typhi isolates**	SLH_01, SLH_10	Clinical isolate	SLH, Manila (CAB Study)	ONT SUP sheared 24-plex	Excluded from the analysis	Excluded from the analysis	Excluded from the analysis

List of benchmark strains (Ty2, Ty42, Ty43) and clinical isolates (SLH_02–SLH_21) included in Run1 (6-plex benchmark sequencing) and Run2 (24-plex field sequencing). Sequencing datasets are distinguished by basecalling mode (SUP or HAC), DNA preparation (sheared or unsheared), and multiplex level (6-plex or 24-plex). Hybrid assemblies were generated using ONT and Illumina data and therefore do not have associated DRA run accessions. Only isolates confirmed as *S.* Typhi by PCR and in silico typing were included in downstream analyses. SUP = Super Accuracy basecalling, HAC = High Accuracy basecalling, SLH = San Lazaro Hospital, CAB = Community-acquired bacteremia, NIID = National Institute of Infectious Diseases.

Supplementary Table S2. Coverage and assembly contiguity of isolates, Run 1 (6-plex)

Strains	Pre-filtered Coverage (×)	Post-filtered Coverage (×)	Mode	DNA Type	Closed Genome (Yes/No)	# contigs
**Ty2**	1252	100	HAC	Sheared	No	2
**Ty2**	1283	100	SUP	Sheared	No	2
**Ty42**	1324	100	HAC	Sheared	No	3
**Ty42**	1351	100	SUP	Sheared	Yes	1
**Ty43**	1584	100	HAC	Sheared	Yes	1
**Ty43**	1623	100	SUP	Sheared	No	6
**Ty2**	234	100	HAC	Unsheared	No	2
**Ty2**	241	100	SUP	Unsheared	No	2
**Ty42**	319	100	HAC	Unsheared	No	5
**Ty42**	330	100	SUP	Unsheared	Yes	1
**Ty43**	73	68	HAC	Unsheared	No	6
**Ty43**	76	70	SUP	Unsheared	No	6

All assemblies were generated using Flye and polished with three rounds of Medaka. "Closed genome" indicates a single circular contig reported by Flye. Pre-filtered coverage = raw sequencing depth estimated from NanoPlot before read filtering with Filtlong. Post-filtered coverage = achieved depth after read filtering and Rasusa downsampling to ~100×. HAC = High Accuracy basecalling; SUP = Super Accuracy basecalling

Supplementary Table S3. Coverage and assembly contiguity of isolates, Run 2 (24-plex)

Strains	Pre-filtered Coverage (×)	Post-filtered Coverage (×)	Mode	DNA Type	Closed Genome (Yes/No)	# contigs
**Ty2**	419	100	SUP	Sheared	No	2
**Ty42**	256	100	SUP	Sheared	Yes	1
**Ty43**	329	100	SUP	Sheared	No	7
**SLH_01**	218	100	SUP	Sheared	No	4
**SLH_02**	482	100	SUP	Sheared	Yes	1
**SLH_03**	475	100	SUP	Sheared	Yes	1
**SLH_04**	475	100	SUP	Sheared	No	5
**SLH_05**	360	100	SUP	Sheared	Yes	1
**SLH_06**	331	100	SUP	Sheared	Yes	1
**SLH_07**	350	100	SUP	Sheared	Yes	1
**SLH_08**	413	100	SUP	Sheared	Yes	1
**SLH_09**	377	100	SUP	Sheared	Yes	1
**SLH_10**	228	100	SUP	Sheared	No	3
**SLH_11**	242	100	SUP	Sheared	Yes	1
**SLH_12**	277	100	SUP	Sheared	No	4
**SLH_13**	443	100	SUP	Sheared	Yes	1
**SLH_14**	311	100	SUP	Sheared	Yes	1
**SLH_15**	534	100	SUP	Sheared	Yes	1
**SLH_16**	420	100	SUP	Sheared	Yes	1
**SLH_17**	249	100	SUP	Sheared	Yes	1
**SLH_18**	244	100	SUP	Sheared	Yes	1
**SLH_19**	328	100	SUP	Sheared	Yes	1
**SLH_20**	338	100	SUP	Sheared	Yes	1
**SLH_21**	136	100	SUP	Sheared	Yes	1

All assemblies were generated using Flye and polished with three rounds of Medaka. "Closed genome" indicates a single circular contig reported by Flye. Pre-filtered coverage = raw sequencing depth estimated from NanoPlot before read filtering with Filtlong. Post-filtered coverage = achieved depth after read filtering and Rasusa downsampling to ~100×. HAC = High Accuracy basecalling; SUP = Super Accuracy basecalling.

Supplementary Table S4. Genotyping, serotyping, MLST, and *in silico* AMR predictions for benchmark and clinical isolates in Run 2 (24-plex)

Strain	ST	Serotype	Genotype	Antigenic profile	O antigen	H1 antigen	*in silico* Mykrobe AMR predictions
**Benchmark strains**							
**Ty2**	1	Typhi	4.1	9:d:-	9	d	No known AMR determinants detected
**Ty42**	2	Typhi	2.3.3	9:d:-	9	d	No known AMR determinants detected
**Ty43**	1	Typhi	4	9:j:-	9	j	No known AMR determinants detected
**SLH clinical isolates**							
**SLH_02,07,12,15,18**	1	Typhi	4.1	9:d:-	9	d	No known AMR determinants detected
**SLH_03,04,05,06,08,09,11,13,14,16,17, 20,21**	1	Typhi	3	9:d:-	9	d	No known AMR determinants detected
**SLH_19**	1	Typhi	3.2.1	9:d:-	9	d	No known AMR determinants detected
**SLH_01, SLH_10**	-	Not Typhi	-	-	-	-	Excluded after *in silico* confirmation of non-Typhi

Genotype (Mykrobe), serotype (SeqSero2), MLST, and in silico AMR predictions for benchmark and clinical isolates sequenced in Run 2. Isolates SLH_01 and SLH_10 were excluded after *in silico* confirmation as non-Typhi. SLH = San Lazaro Hospital

Supplementary Table S5. MOB-suite plasmid screening for benchmark and SLH field-run assemblies

Strain	Dataset	Assembly mode	Contigs screened	MOB-suite plasmid detected	Plasmid-associated markers detected	Interpretation
**Ty2**	ONT long read + Illumina short read	Hybrid	2	No	No	507 kb secondary contig classified as chromosomal
**Ty2**	Illumina MiSeq	Illumina	108	No	No	Short-read draft assembly; all contigs classified as chromosomal; no plasmid-classified contig detected
**Ty2**	Benchmark 6-plex	SUP_Sheared	2	No	No	531 kb secondary contig classified as chromosomal
**Ty2**	Benchmark 6-plex	SUP_Unsheared	2	No	No	507 kb secondary contig classified as chromosomal
**Ty42**	ONT long read + Illumina short read	Hybrid	1	No	No	Complete chromosomal assembly; no plasmid-classified contig detected
**Ty42**	Illumina MiSeq	Illumina	139	No	No	Short-read draft assembly; all contigs classified as chromosomal; no plasmid-classified contig detected
**Ty42**	Benchmark 6-plex	SUP_Sheared	1	No	No	Complete chromosomal assembly; no plasmid-classified contig detected
**Ty42**	Benchmark 6-plex	SUP_Unsheared	1	No	No	Complete chromosomal assembly; no plasmid-classified contig detected
**Ty43**	ONT long read + Illumina short read	Hybrid	7	No	No	Fragmented chromosomal assembly; no plasmid-classified contig detected

**Ty43**	Illumina MiSeq	Illumina	165	No	No	Short-read draft assembly; all contigs classified as chromosomal; no plasmid-classified contig detected
**Ty43**	Benchmark 6-plex	SUP_Sheared	6	No	No	Fragmented chromosomal assembly; no plasmid-classified contig detected
**Ty43**	Benchmark 6-plex	SUP_Unsheared	6	No	No	Fragmented chromosomal assembly; no plasmid-classified contig detected
**SLH_02**	Field run 24-plex	SUP_Sheared	1	No	No	Complete chromosomal assembly; no plasmid-classified contig detected
**SLH_03**	Field run 24-plex	SUP_Sheared	1	No	No	Complete chromosomal assembly; no plasmid-classified contig detected
**SLH_04**	Field run 24-plex	SUP_Sheared	5	No	IncP replicon; MOBH/MOBP relaxase markers	Plasmid-associated markers detected on contigs classified as chromosomal; not interpreted as reconstructed plasmids
**SLH_05**	Field run 24-plex	SUP_Sheared	1	No	No	Complete chromosomal assembly; no plasmid-classified contig detected
**SLH_06**	Field run 24-plex	SUP_Sheared	1	No	No	Complete chromosomal assembly; no plasmid-classified contig detected
**SLH_07**	Field run 24-plex	SUP_Sheared	1	No	No	Complete chromosomal assembly; no plasmid-classified contig detected
**SLH_08**	Field run 24-plex	SUP_Sheared	1	No	No	Complete chromosomal assembly; no plasmid-classified contig detected
**SLH_09**	Field run 24-plex	SUP_Sheared	1	No	No	Complete chromosomal assembly; no plasmid-classified contig detected
**SLH_11**	Field run 24-plex	SUP_Sheared	1	No	No	Complete chromosomal assembly; no plasmid-classified contig detected
**SLH_12**	Field run 24-plex	SUP_Sheared	4	No	No	Fragmented chromosomal assembly; no plasmid-classified contig detected
**SLH_13**	Field run 24-plex	SUP_Sheared	1	No	No	Complete chromosomal assembly; no plasmid-classified contig detected
**SLH_14**	Field run 24-plex	SUP_Sheared	1	No	No	Complete chromosomal assembly; no plasmid-classified contig detected

**SLH_15**	Field run 24-plex	SUP_Sheared	1	No	No	Complete chromosomal assembly; no plasmid-classified contig detected
**SLH_16**	Field run 24-plex	SUP_Sheared	1	No	No	Complete chromosomal assembly; no plasmid-classified contig detected
**SLH_17**	Field run 24-plex	SUP_Sheared	1	No	No	Complete chromosomal assembly; no plasmid-classified contig detected
**SLH_18**	Field run 24-plex	SUP_Sheared	1	No	No	Complete chromosomal assembly; no plasmid-classified contig detected
**SLH_19**	Field run 24-plex	SUP_Sheared	1	No	No	Complete chromosomal assembly; no plasmid-classified contig detected
**SLH_20**	Field run 24-plex	SUP_Sheared	1	No	No	Complete chromosomal assembly; no plasmid-classified contig detected
**SLH_21**	Field run 24-plex	SUP_Sheared	1	No	No	Complete chromosomal assembly; no plasmid-classified contig detected

Benchmark strain assemblies from Run 1 (6-plex) and SLH field-run assemblies from Run 2 (24-plex) were screened using MOB-suite to identify plasmid-classified contigs and plasmid-associated markers. Benchmark assemblies included Illumina short-read assemblies, hybrid assemblies generated using ONT long reads and Illumina short reads, SUP-sheared ONT-only assemblies generated from mechanically sheared DNA, and SUP-unsheared ONT-only assemblies generated from unsheared DNA for Ty2, Ty42, and Ty43. HAC assemblies were not included in plasmid screening. SLH field-run assemblies were generated only from mechanically sheared DNA and were therefore screened descriptively rather than used to compare plasmid recovery across DNA preparation methods. SUP = Super Accuracy basecalling; HAC = High Accuracy basecalling; ONT = Oxford Nanopore Technologies, SLH = San Lazaro Hospital. Isolates SLH_01 and SLH_10 were excluded following non-Typhi confirmation.

Supplementary Table S6. Structural assembly metrics for Run 2 (24-Plex) ONT assemblies

Sample	Genome Fraction (%)	Misassemblies	Error rate per 100 kb	NGA50 (bp)
**Ty2**	99.99	6	16.41	1184425
**Ty42**	100	2	0	4761719
**Ty43**	98.78	5	0.84	1117655
**SLH_02**	99.82	13	6.36	641743
**SLH_03**	99.17	13	8.11	662966
**SLH_04**	99.20	17	8.97	1214983
**SLH_05**	99.24	12	8.67	667191
**SLH_06**	99.26	15	9.02	641743
**SLH_07**	99.86	11	6.12	1305377
**SLH_08**	99.15	11	8.38	1148993
**SLH_09**	99.18	12	8.59	667198
**SLH_11**	99.31	8	9.53	662966
**SLH_12**	99.41	15	6.53	1215267
**SLH_13**	99.15	14	8.95	661685
**SLH_14**	99.26	13	8.87	654966
**SLH_15**	99.79	7	6.26	1215268
**SLH_16**	99.15	14	8.85	636357
**SLH_17**	99.15	16	8.65	792903
**SLH_18**	99.79	11	7.09	779414
**SLH_19**	99.10	20	11.21	745492
**SLH_20**	99.17	10	8.88	661667
**SLH_21**	99.18	13	8.77	667167

QUAST structural metrics for 24-plex ONT assemblies. Benchmark strains (Ty2, Ty42, Ty43) were evaluated against their respective strain-specific hybrid assemblies. Clinical SLH isolates were evaluated against the *Salmonella enterica* serovar Typhi Ty2 reference genome (NC_004631.1) for consistency across samples. Non-Typhi isolates (SLH_01, SLH_10) were excluded. SLH = San Lazaro Hospital.

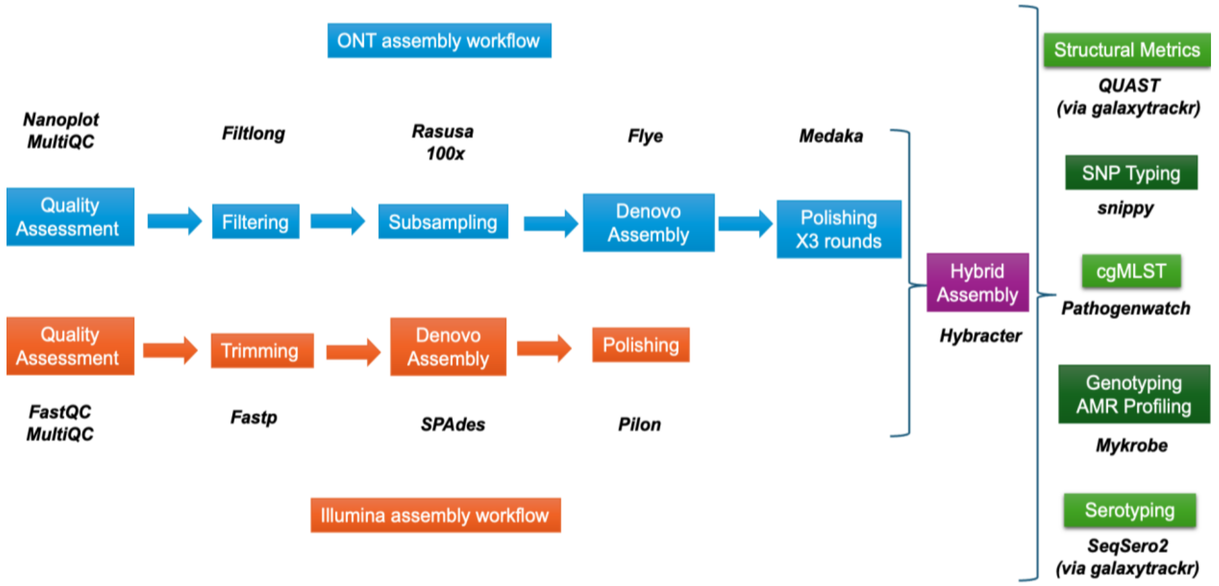

Supplementary Figure S1. Overview of sequencing and analysis workflow.
Diagram summarizing the analytical pipeline for ONT and Illumina datasets. Strain-specific hybrid assemblies were generated by combining Illumina reads with high-quality ONT long reads using Hybracter. All genome assemblies and SNP analysis (Snippy), genotyping, and AMR prediction (Mykrobe) were performed via command-line workflows. Structural accuracy assessment (QUAST) and serotyping (SeqSero2) were conducted via the GalaxyTrakr cloud platform, while cgMLST profiling was performed using Pathogenwatch, see methods for details.

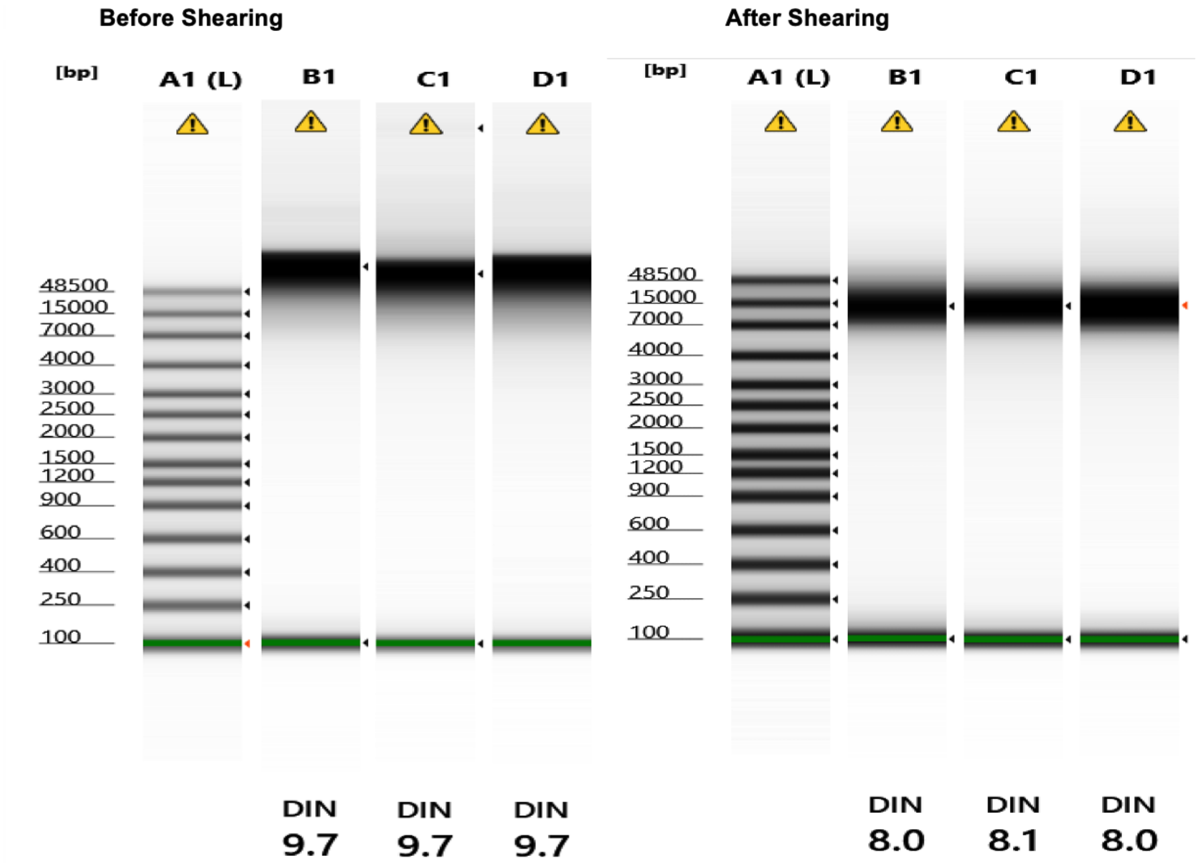

Supplementary Figure S2. DNA fragmentation profile before and after shearing
High-molecular-weight gDNA was evaluated using the Agilent DNA ScreenTape platform before and after shearing. Panels: **Left:** High–molecular–weight gDNA before shearing. **Right:** Fragment size distribution after Covaris g-TUBE™ shearing. A1 (L): DNA ladder; B1: Ty2; C1: Ty42; D1: Ty43. DIN = DNA Integrity Number (maximum score: 10).

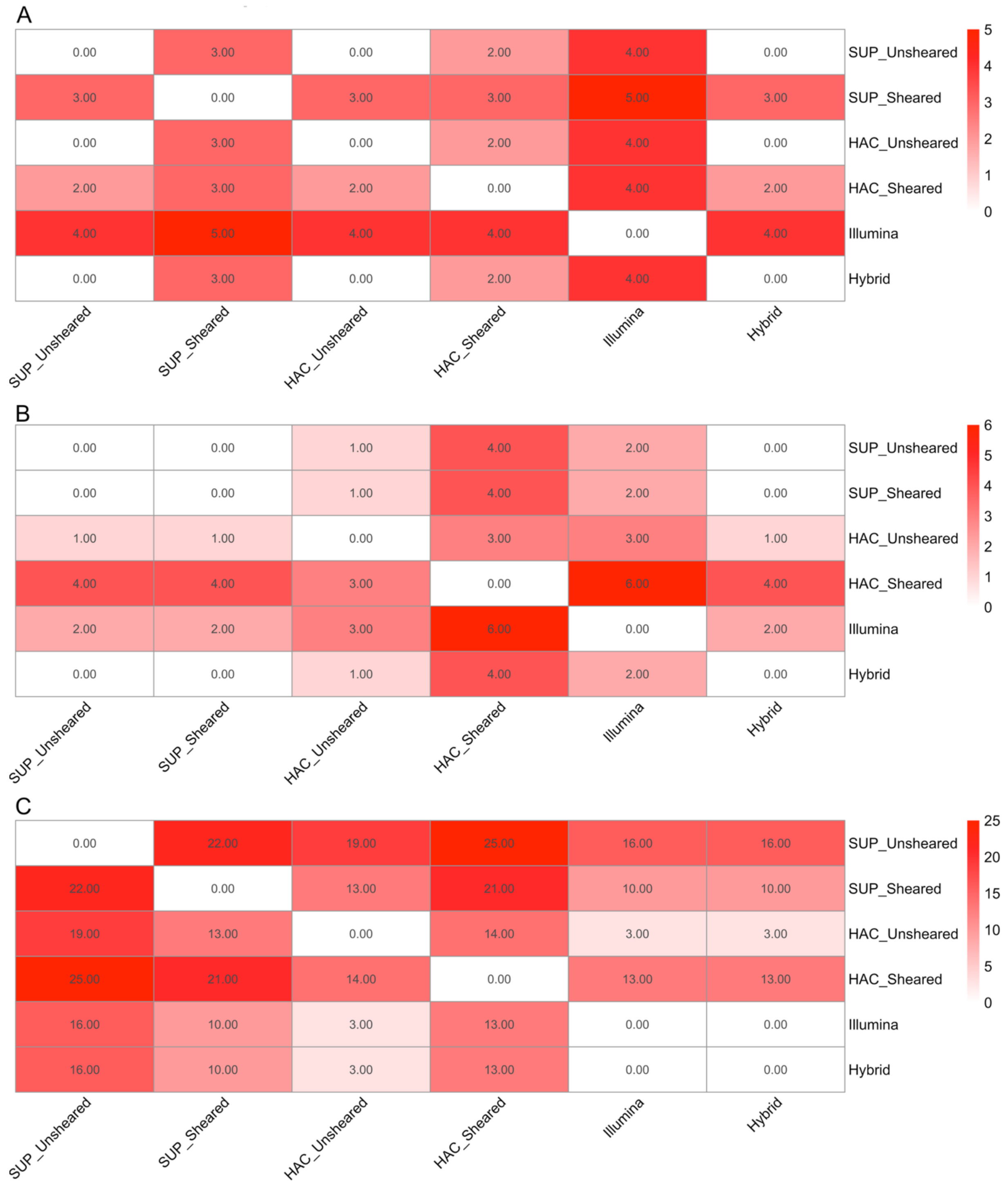

Supplementary Figure S3. Pairwise SNV distance heatmaps for benchmark strains, Run 1 (6-plex)

Heatmaps showing pairwise SNV distances among assemblies for (A) SNV distances between Ty2 (6-plex) and Ty2 hybrid reference, (B) SNV distances between Ty42 (6-plex) and Ty42 hybrid reference, and (C) SNV distances between Ty43 (6-plex) and Ty43 hybrid reference. Distances were calculated using Snippy on core genome alignments.

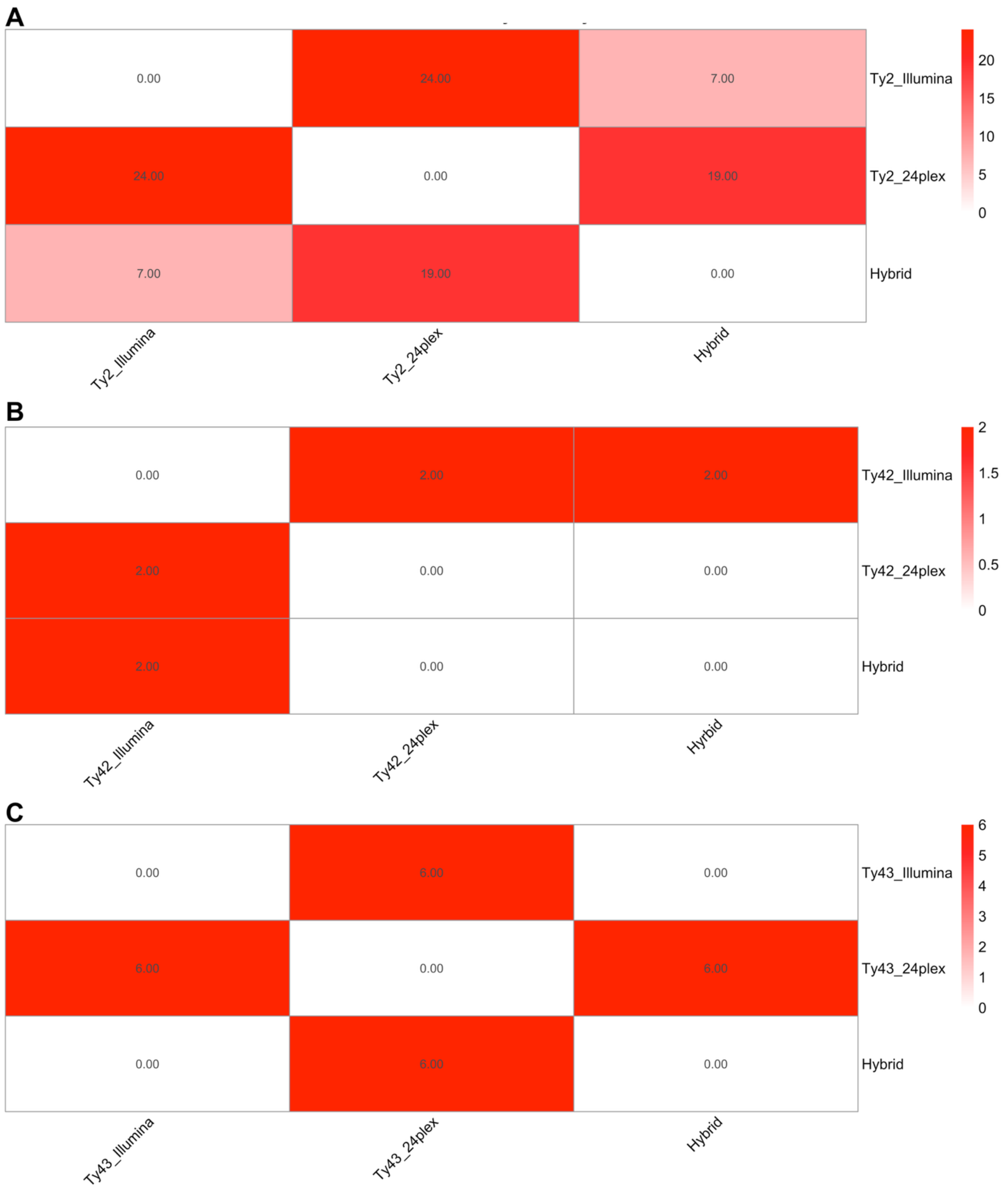

Supplementary Figure S4. Pairwise SNV distance heatmaps for benchmark strains, Run 2 (24-Plex)

Heatmaps showing pairwise SNV distances among assemblies for (A) SNV distances between Ty2 (24-plex) and Ty2 hybrid reference, (B) SNV distances between Ty42 (24-plex) and Ty42 hybrid reference, and (C) SNV distances between Ty43 (24-plex) and Ty43 hybrid reference. Distances were calculated using Snippy on core genome alignments.

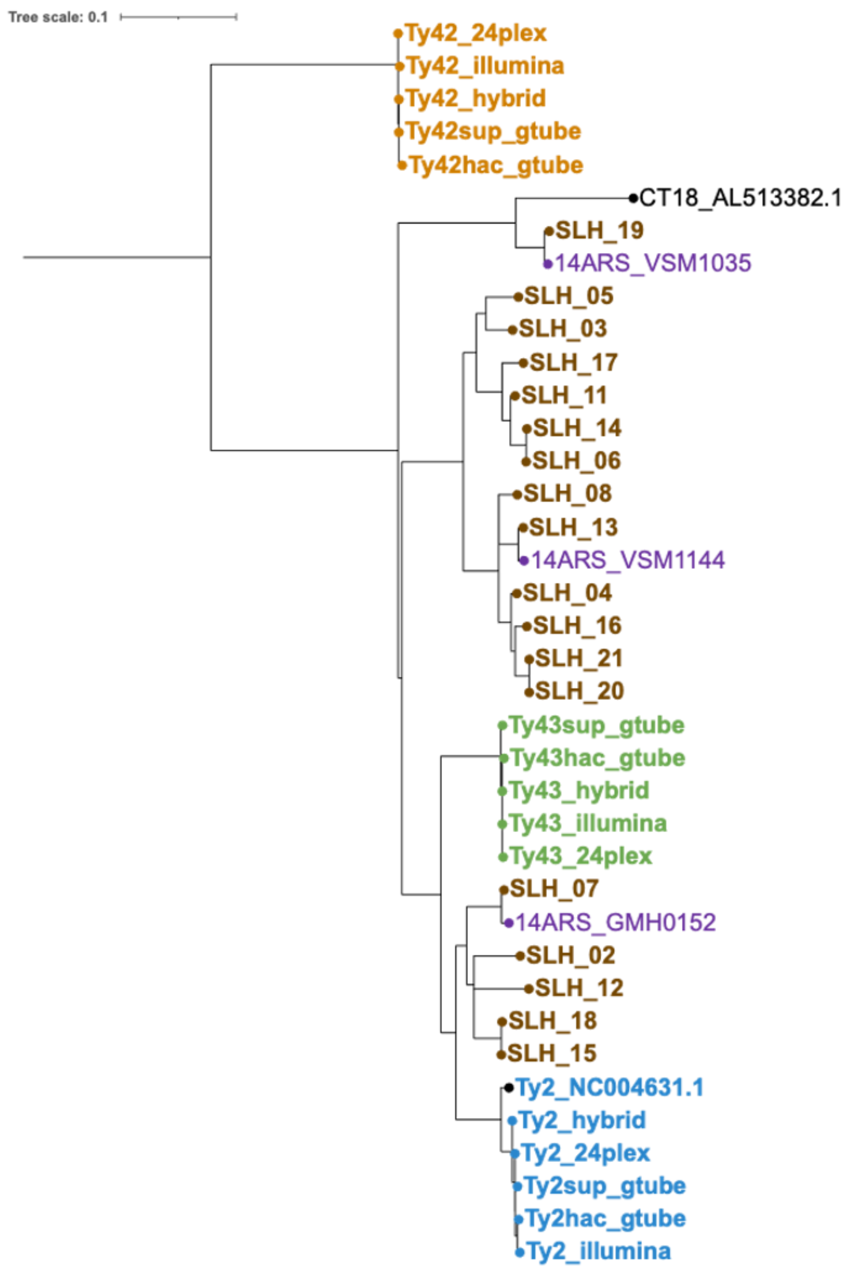

Supplementary Figure S5: Recombination-filtered maximum likelihood (ML) phylogeny of benchmark and clinical *S.* Typhi isolates

ML phylogeny generated from core SNP alignments using Ty2_NC004631.1 as the reference (4.79 Mb; GTR+G model; 1,000 ultrafast bootstrap replicates; midpoint rooted). Recombinant regions were identified using Gubbins and masked before phylogenetic inference. The tree compares ONT, Illumina, and hybrid assemblies from three benchmark strains (Ty2, Ty42, Ty43) from the 6-plex and 24-plex runs, 18 SLH clinical isolates, and three publicly available Philippine genomes (14ARS collection), with CT18_AL513382.1 and Ty2_NC004631.1 included for phylogenetic context. SLH_09 was excluded from the displayed tree because it had an identical recombination-filtered core-SNP profile to SLH_05; available sample metadata did not indicate duplicate sampling. Node colors indicate sample origin: Ty2 (blue), Ty42 (orange), Ty43 (green), SLH clinical isolates (brown), Philippine public genomes (purple), and reference genomes (black). The scale bar represents the number of nucleotide substitutions per site in the recombination-filtered core-SNP alignment.
